# Supplementary figures and images for: The impact of global and local Polynesian genetic ancestry on complex traits in Native Hawaiians
Source: PLoS Genet. 2021 Feb 11;17(2):e1009273. doi: 10.1371/journal.pgen.1009273 (PMC7877570; doi:10.1371/journal.pgen.1009273)

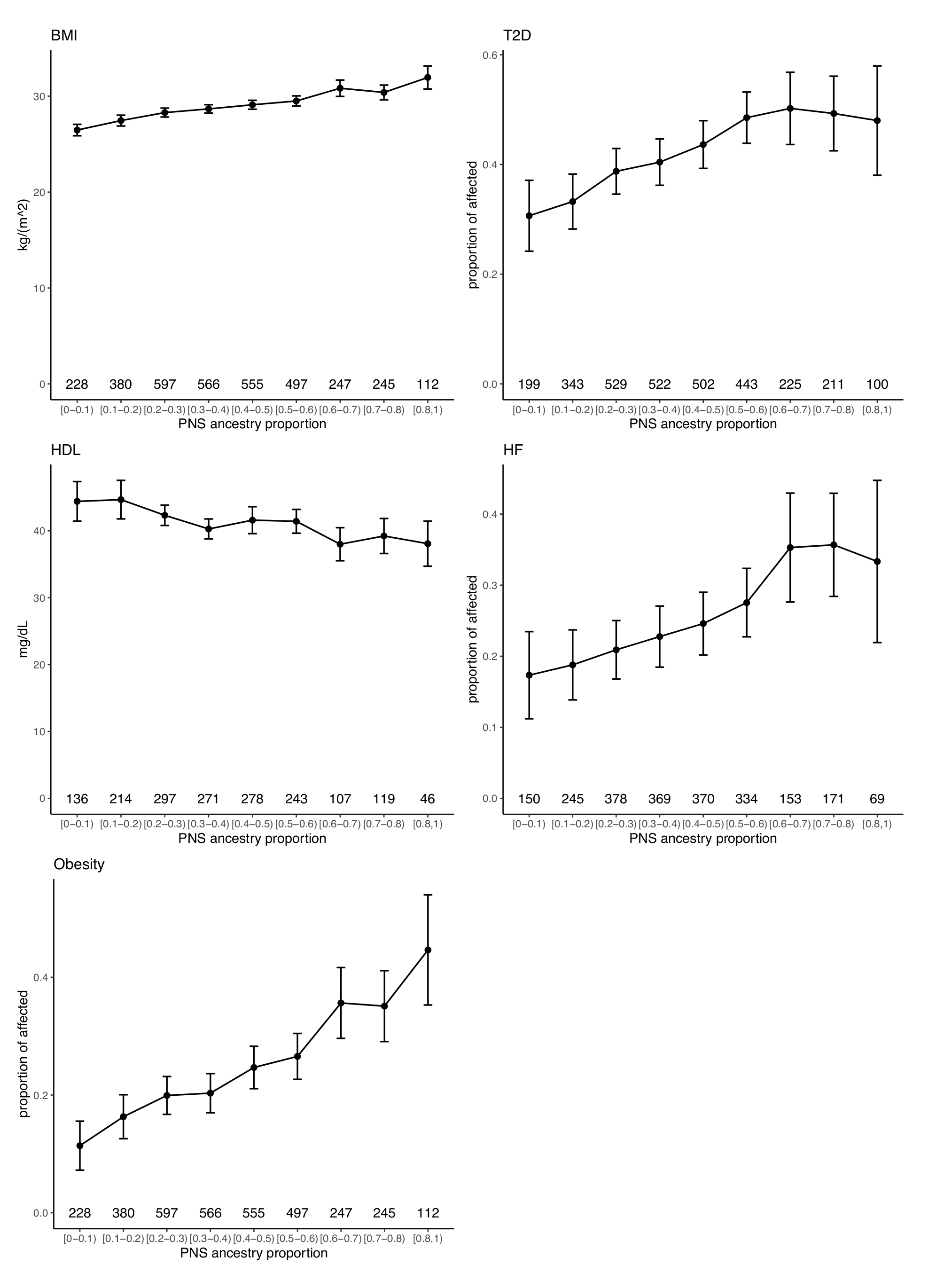

Supplement: S1 Fig — For each of five traits that we found significant association with PNS ancestry, we show the mean and standard error of untransformed trait value (for quantitative trait) or proportion of affected (for dichotomous) trait as function of bins of PNS ancestry. The sample size for individual available is given above each bin. (TIF) [file pgen.1009273.s001.tif]

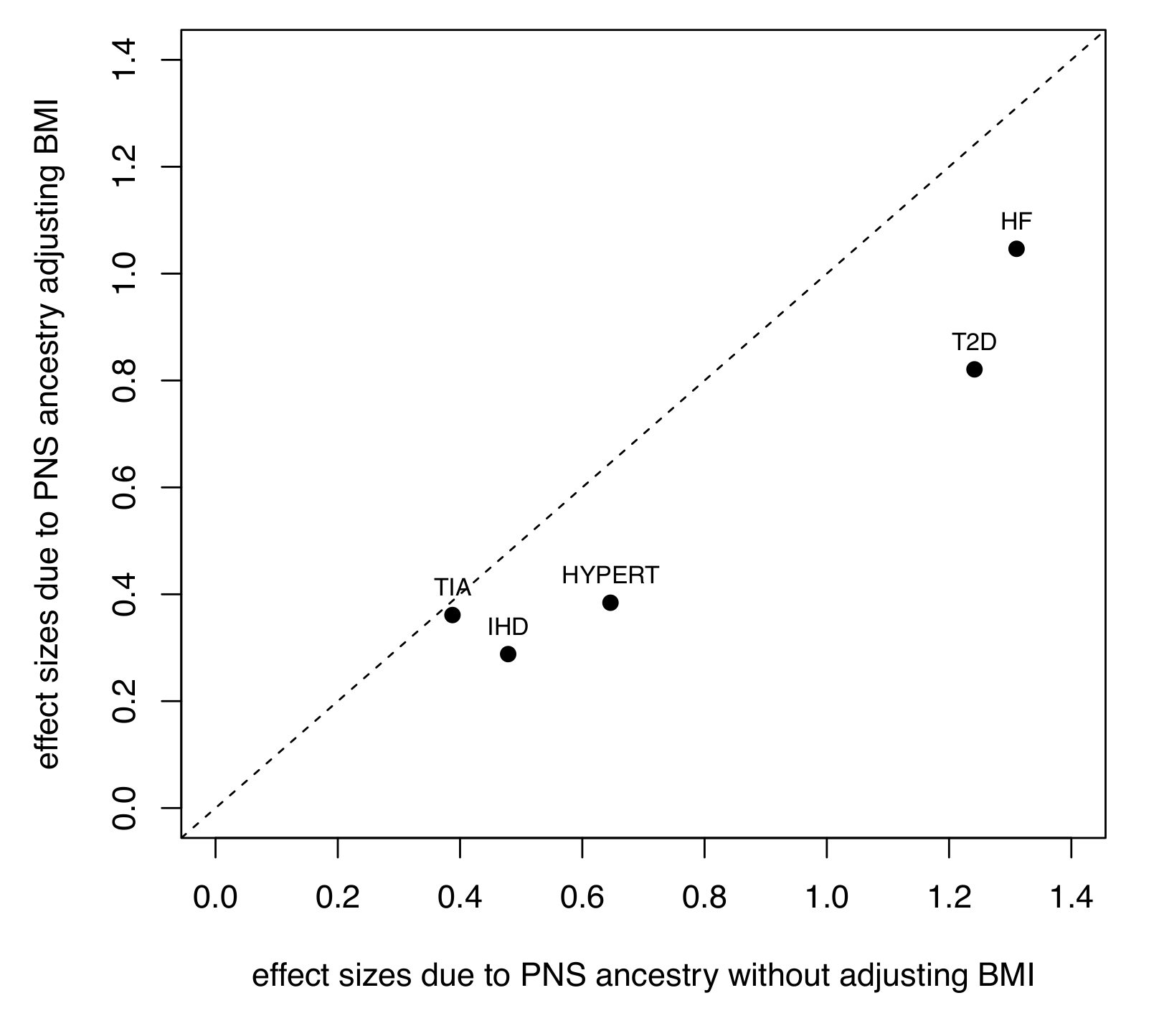

Supplement: S2 Fig — Across the binary traits tested, even if the effect attributable to PNS ancestry is not significant, the effect sizes are lowered if accounting for BMI, suggesting at least part of the excess risk for these traits among Native Hawaiians are mediated through higher BMI associated with the ancestry. Hyperlipidemia was excluded because BMI is not associated with the disease risk in univariate regression model. HF, heart failure; HYPERT, hypertension; IHD, ischemic heart disease; T2D, type-2 diabetes; TIA, stroke and transient ischemic attack. (TIF) [file pgen.1009273.s002.tif]

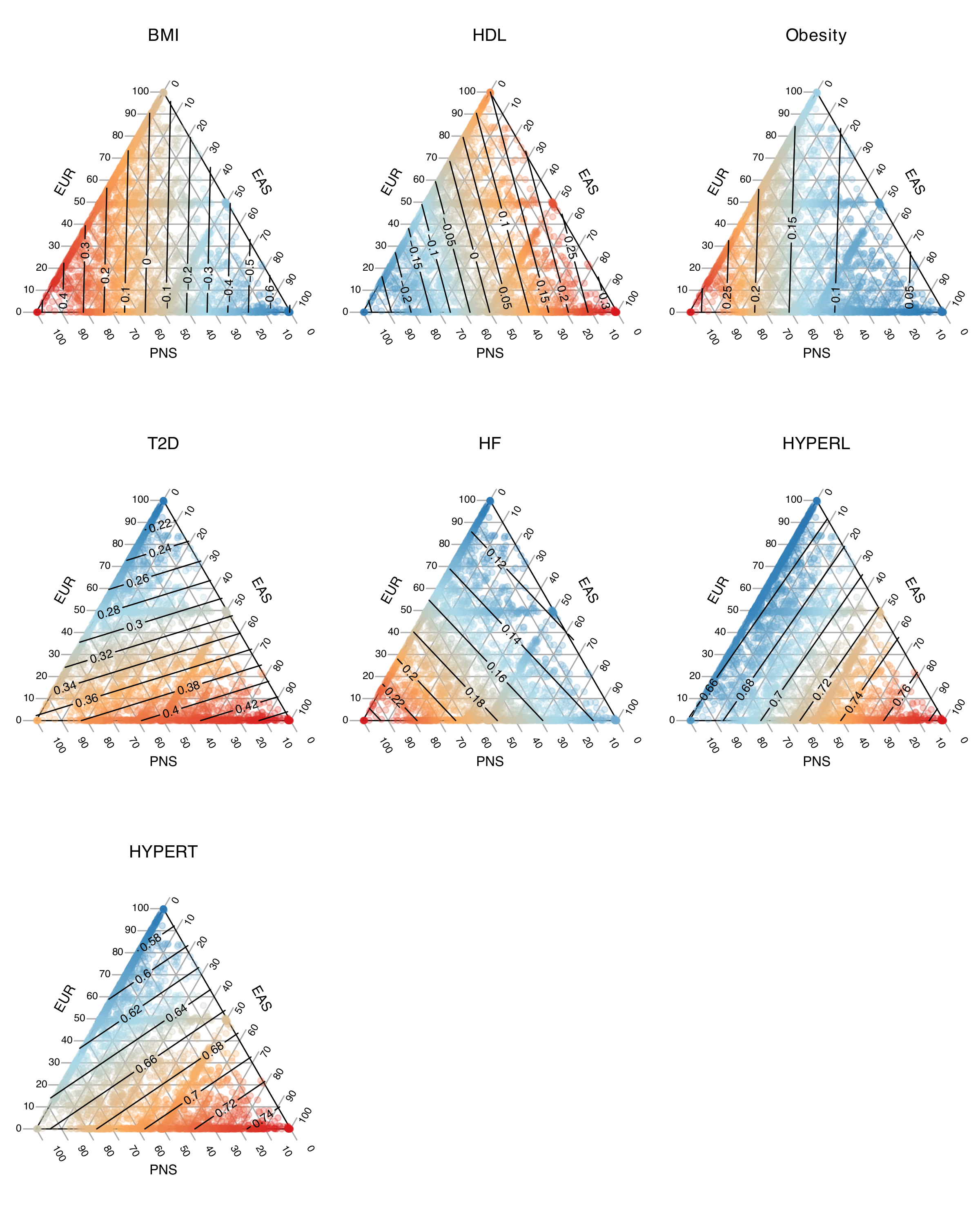

Supplement: S3 Fig — For the seven traits in Table 1 in which as least one component of ancestry showed significant association, we estimated the fitted trait value (in units of s.d. for quantitative traits BMI and HDL) or probability of being affected (for dichotomous traits Obesity, T2D, HF, HYPERL, and HYPERT) for each person in our dataset, given their estimated proportion of ancestry. For simplicity, we removed individuals with estimated AFR ancestry > 0.05, and re-normalized the EAS, EUR, and PNS ancestry to sum to 1 in the remaining individual. We used Model 2 in S1 Table for BMI and S5 Table for HDL to obtain predicted phenotype residual in units of standard deviation. For dichotomous traits, we used Model 2 in S9–S13 Tables for Obesity, T2D, HF, HYPERL, and HYPERT, respectively, and converted the fitted log-odds to probability of being affected. Because the covariates are included in the model rather than being regressed out in quantitative traits, we assumed fixed values of age = 50, BMI = 30 (except for obesity), sex = male, and education level = college graduates. Contour plots are shown labeling in each plot to display the fitted trait value or probability of being affected. (TIF) [file pgen.1009273.s003.tif]

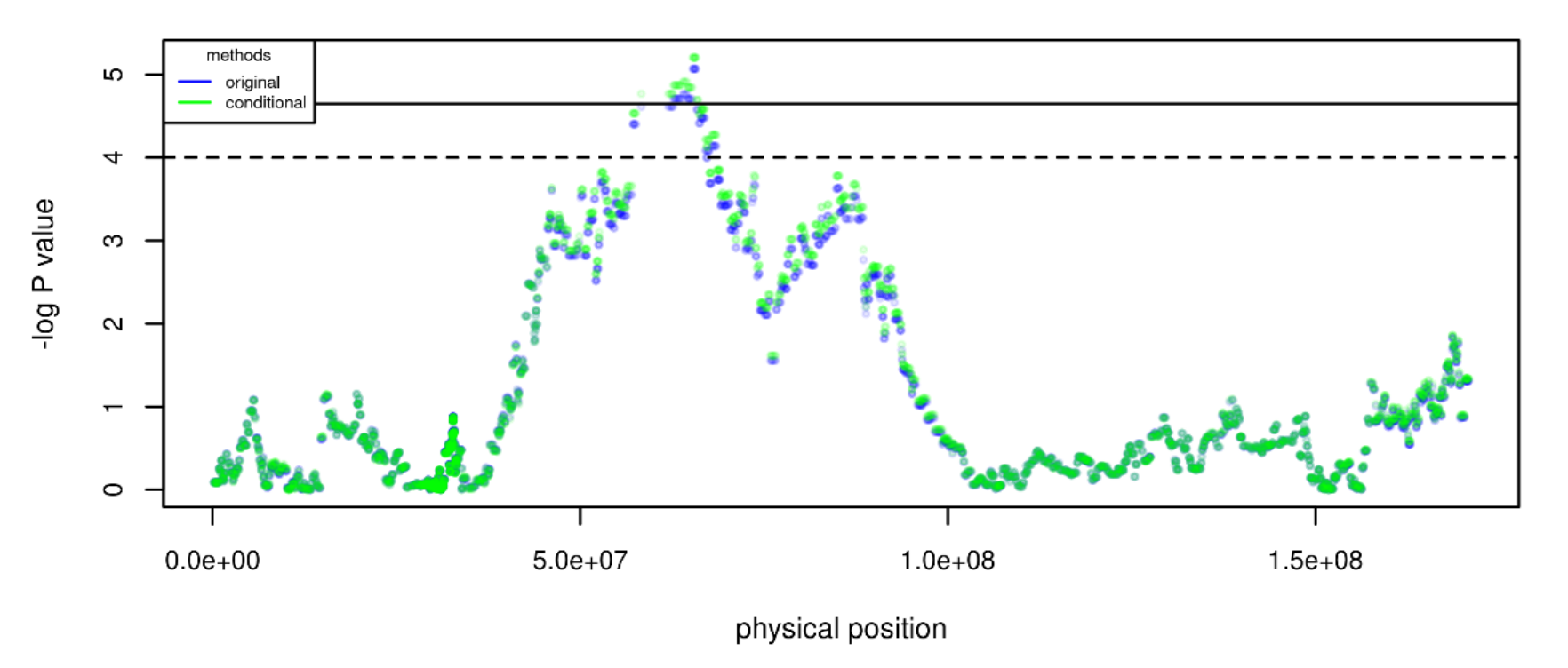

Supplement: S4 Fig — Green and blue colors denote SNP level P-value in association testing with and without, respectively, conditioning on known GWAS variants. (TIF) [file pgen.1009273.s004.tif]

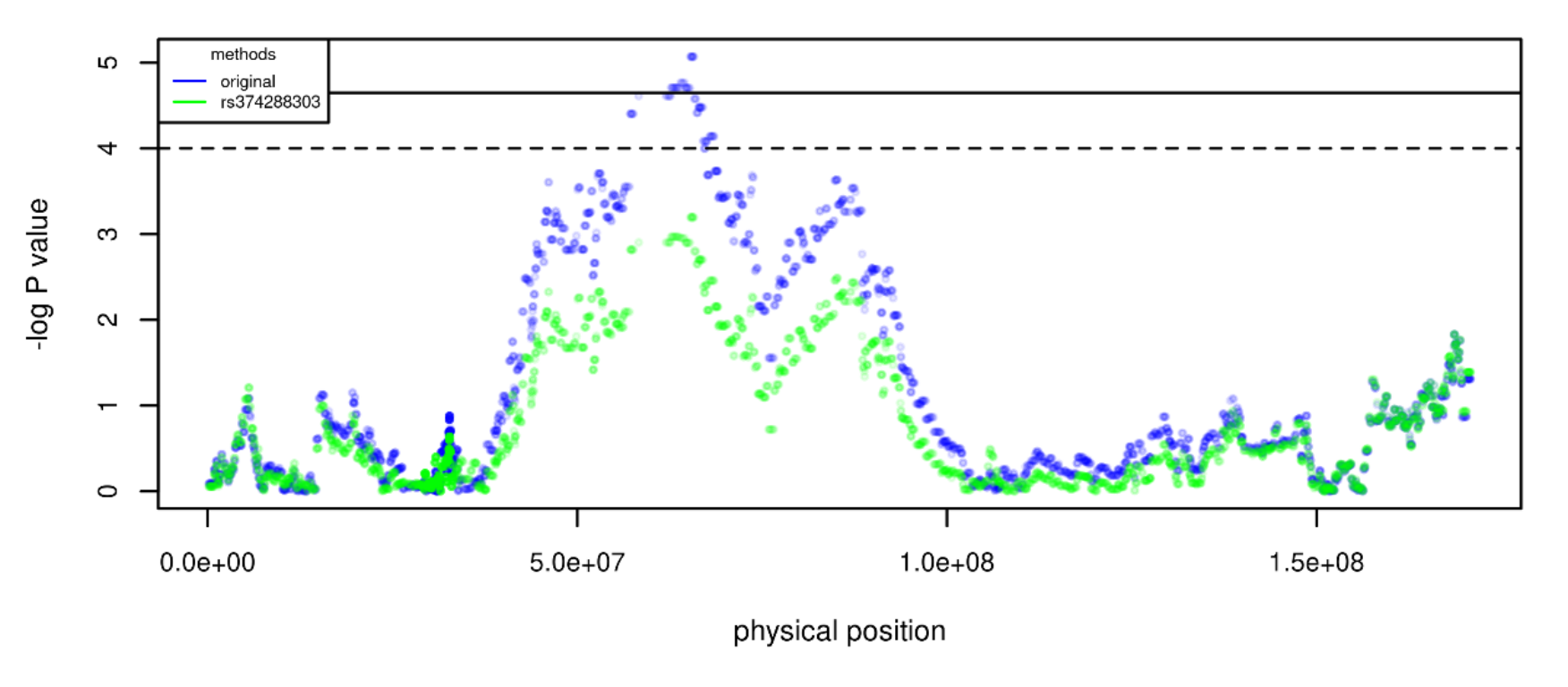

Supplement: S5 Fig — The originally reported admixture signal (blue) can be explained by the conditioned variant (green), suggesting that these single variants might be novel variants associated with these traits. (TIF) [file pgen.1009273.s005.tif]

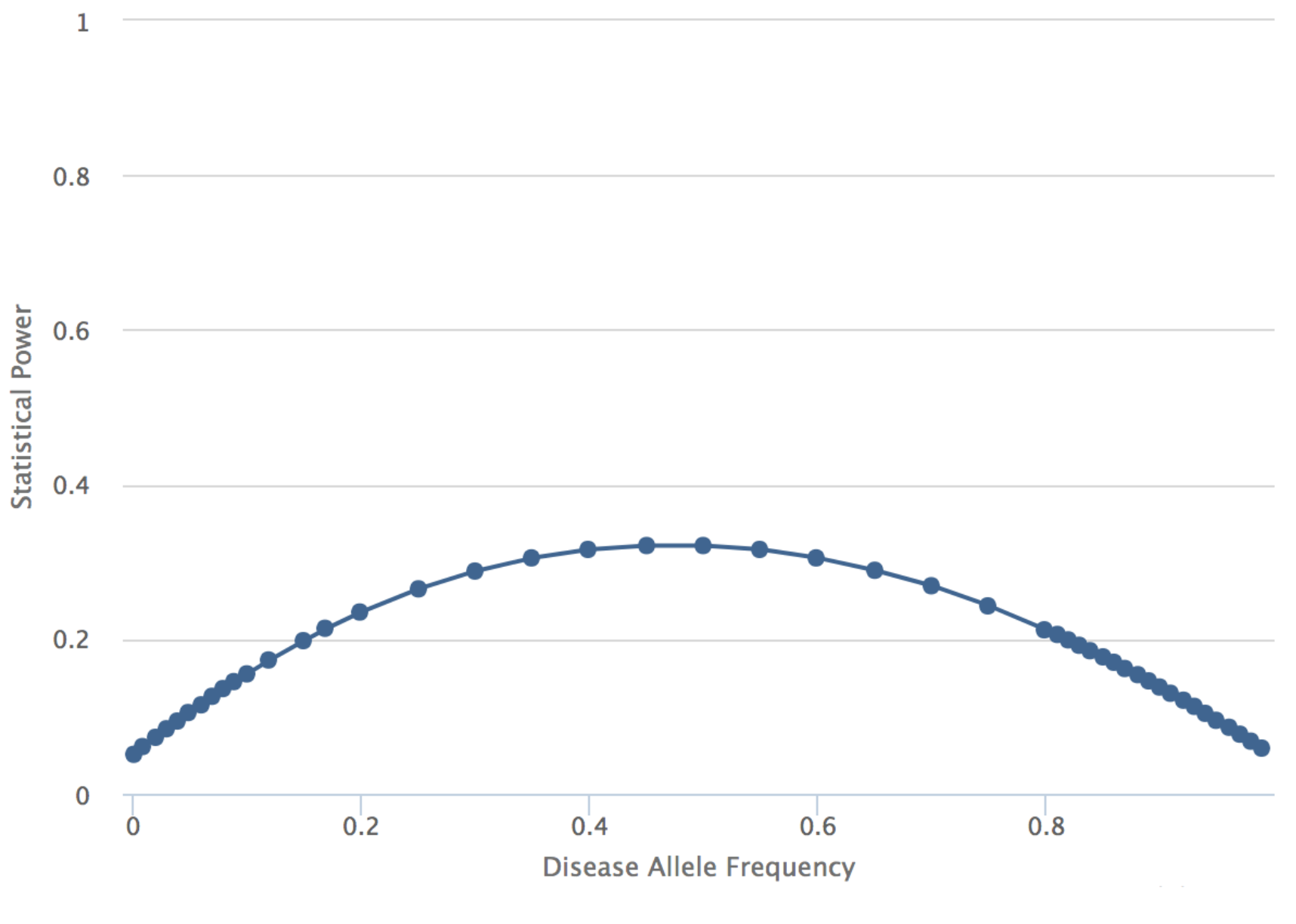

Supplement: S6 Fig — We estimated the power to replicate the top signal (rs370140172) from single variant analysis with T2D in the Samoan cohort, using GAS power calculator (http://csg.sph.umich.edu/abecasis/cats/gas_power_calculator/index.html). The prevalence rate of T2D in Samoans set as 17.1%, which was the value averaged over the reported values in both sex [1]. The number of cases (N = 475) and controls (N = 2377) were set to the observed sample size in Samoans. The genotype relative risk was set to estimated OR (1.096) from MEC-NH. (TIF) [file pgen.1009273.s006.tif]

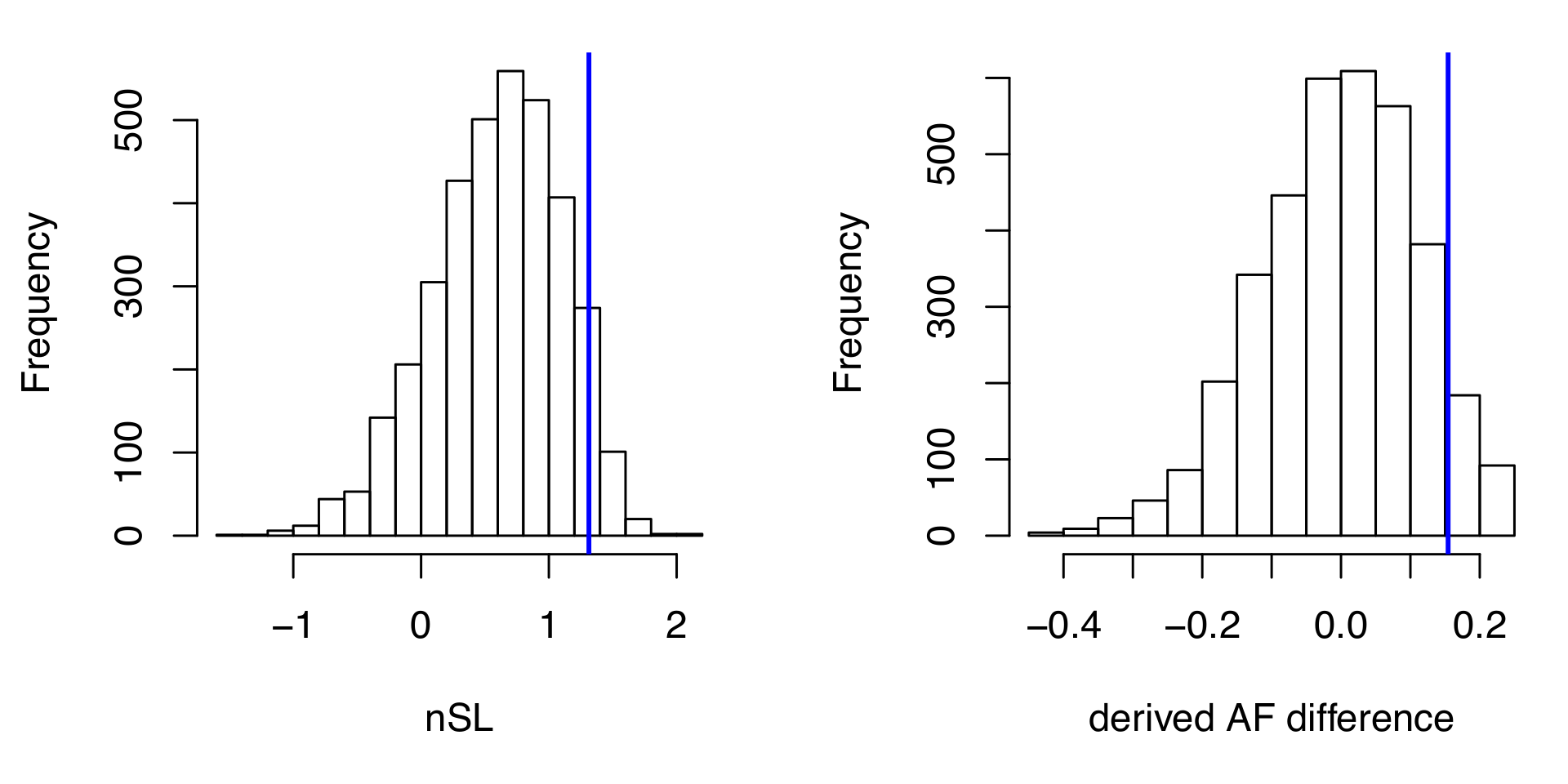

Supplement: S7 Fig — We selected ~44,000 variants across the genome from imputed data matched to rs370140172 by derived allele frequency in MEC-NH and by imputation quality (Methods). We compared the nSL statistics (left) and the difference in frequency of the derived allele between MEC-NH and Samoans (right) at rs370140172 (denoted by the blue vertical line) to the null distribution based on the ~44,000 matched SNP. The evidence of selection for rs370140172 is marginally insignificant by either haplotype length (P = 0.067) or allele frequency differentiation (P = 0.076). (TIF) [file pgen.1009273.s007.tif]

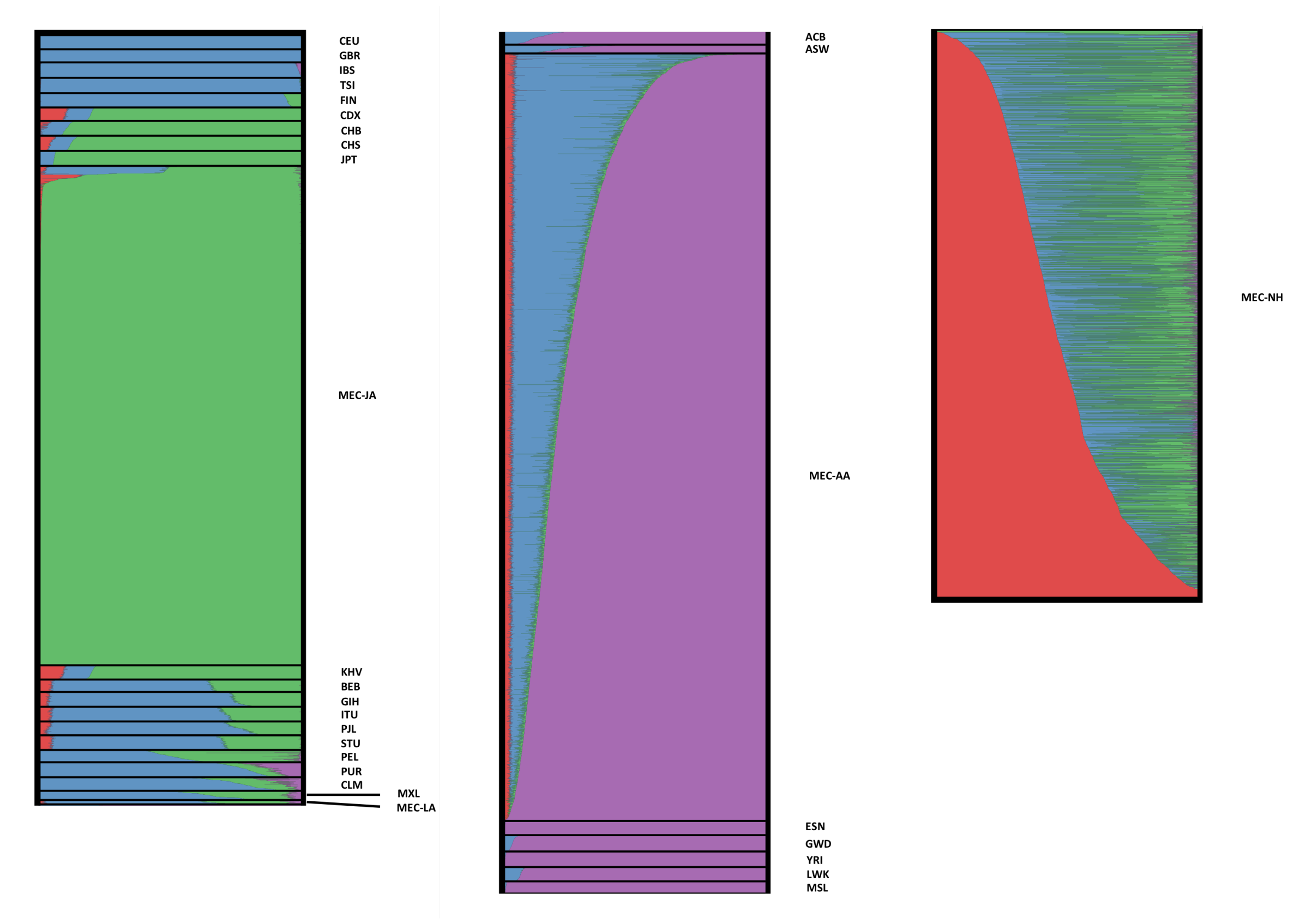

Supplement: S8 Fig — 3,465 MEC Japanese (MEC-JA), 30 MEC Latinos (MEC-LA), 5,325 MEC African Americans (MEC-AA), and 3,940 MEC Native Hawaiians (MEC-NH) were merged with the 1000 Genomes Project populations. At K = 4 we identified an ancestral component (colored red) that are found largely in Native Hawaiians, presumed to be the Polynesian ancestry. (TIF) [file pgen.1009273.s008.tif]

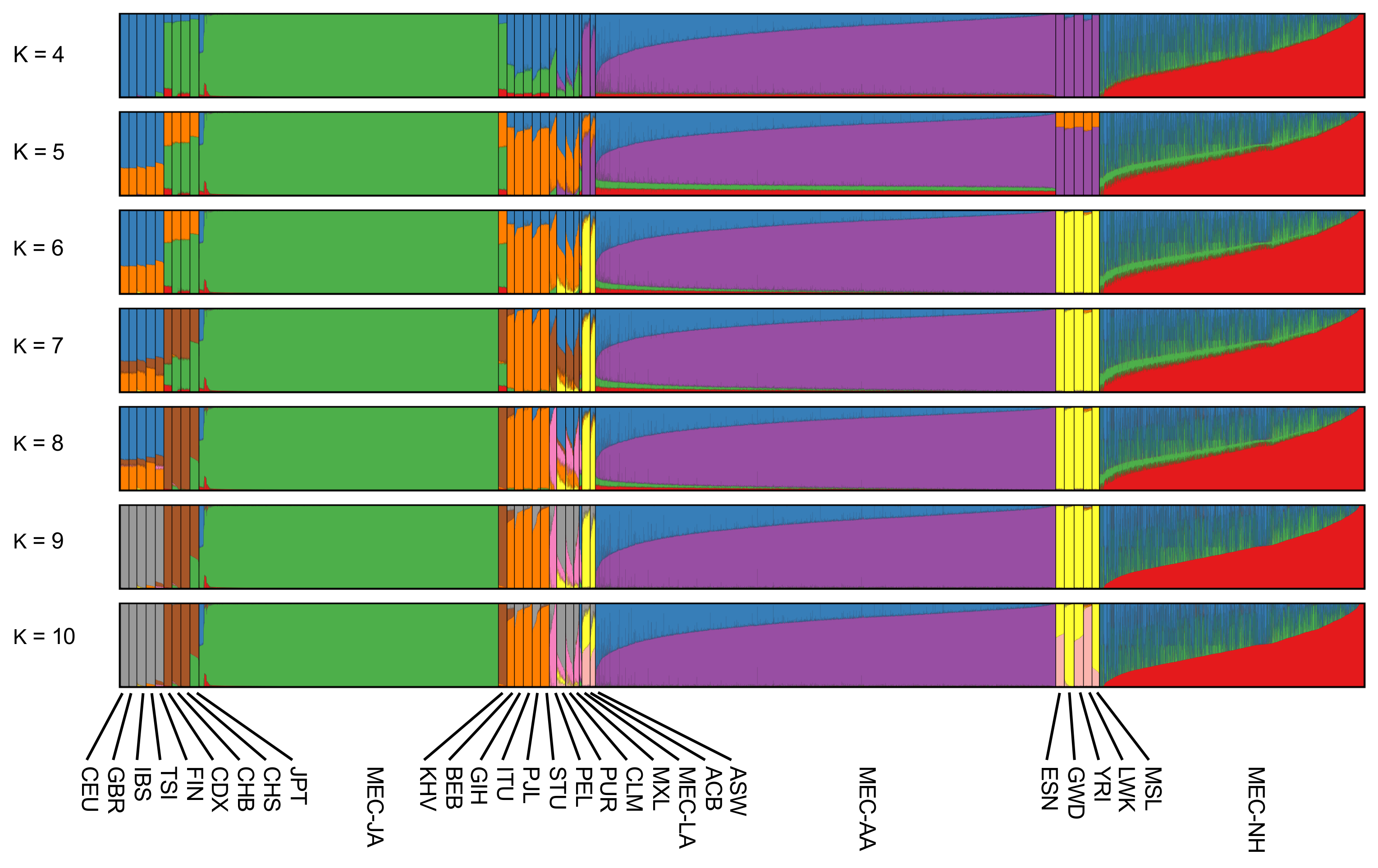

Supplement: S9 Fig — At each K, ancestry proportions from the replicate (out of five) with highest estimated likelihood output by ADMIXTURE were visualized using Pong. Notably, the inferred proportion of PNS component in Native Hawaiians (red component at K = 4) remains stable across higher K. (TIF) [file pgen.1009273.s009.tif]

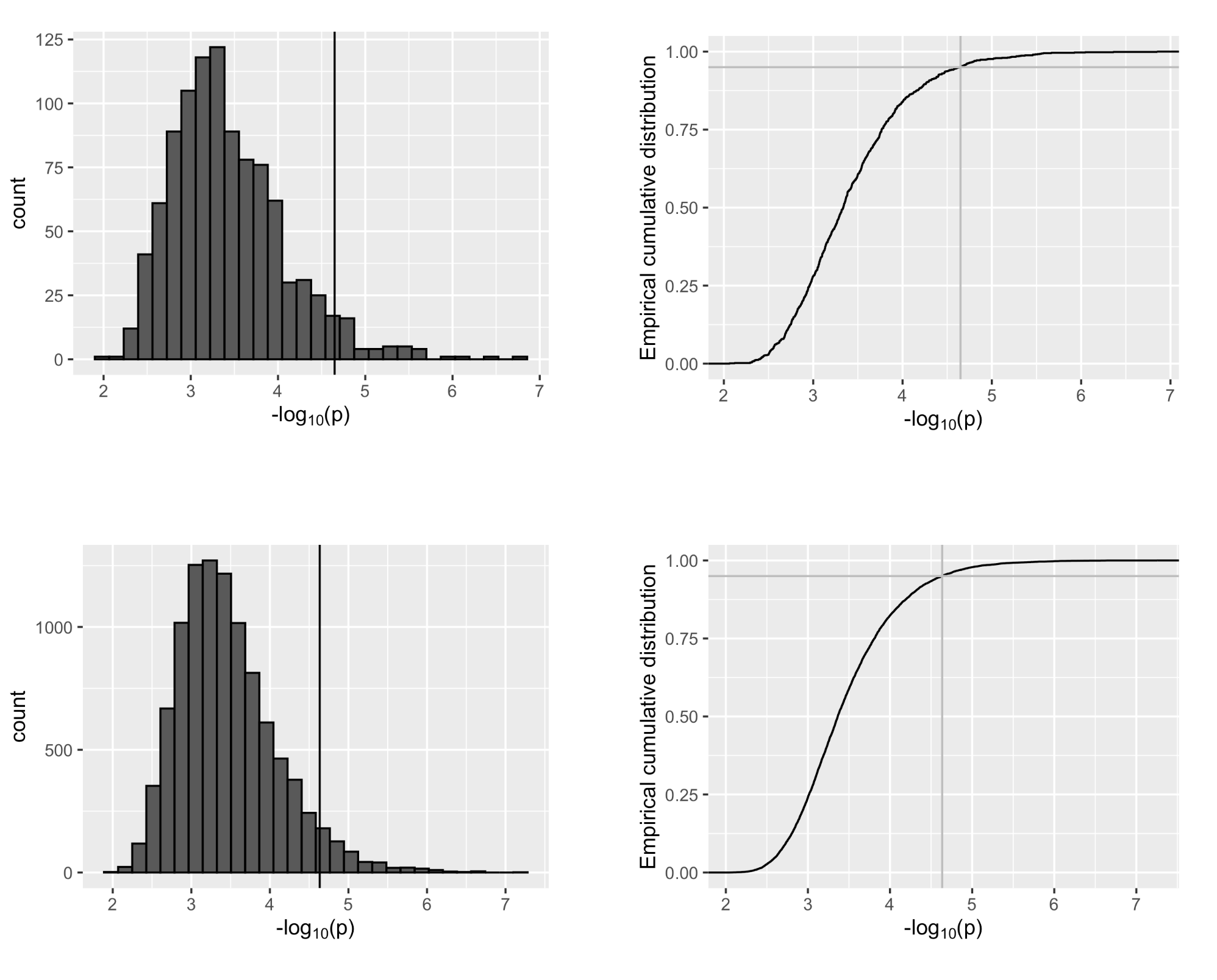

Supplement: S10 Fig — We used 1,000 runs of genome-wide permutation (top) or 10,000 runs of simulation of test statistics using STEAM (bottom) to determine the distribution of admixture mapping test statistics under the null hypothesis given the correlation structure of estimated local ancestry in MEC Native Hawaiians. The significance threshold was then set as the P-value threshold in which we would obtain a 5% false discovery rate. The threshold was 2.24x10^-5^ using permutation, or 2.28x10^-5^ using STEAM. We thus adopt a threshold of 2.2x10^-5^ for our study (Methods). (TIF) [file pgen.1009273.s010.tif]
