# Supplementary material for: The impact of global and local Polynesian genetic ancestry on complex traits in Native Hawaiians
Source: PLoS Genet. 2021 Feb 11;17(2):e1009273. doi: 10.1371/journal.pgen.1009273 (PMC7877570; doi:10.1371/journal.pgen.1009273)
Supplement: S1 Table — Model 1 models the non-genetic covariates according to the heuristic described in the Methods. The residual from model 1 is then inverse normalized and tested in model 2. Models 1A and 2A repeats the procedure but included quintiles of nSES levels in a mixed effect model (Methods); in this case, the R2 in Model 1A reported include both the fixed and the random effect. * edu4 was a binary variable created from the original categorical variable of education status by grouping levels 1,2,3 and coded 0, while education status level 4 was coded as 1. This was done because there were no significant associations between education levels 1 through 3 and BMI. See S21 Table for description of these education levels. (DOCX) [file pgen.1009273.s011.docx]

**S1 Table: Details of the association statistics of the covariates and global ancestries of BMI.**

| Model 1: linear regression between BMI and covariates | | | | | | | |
| --- | --- | --- | --- | --- | --- | --- | --- |
| variables | | estimate | std. error | t | p | R^2^ | df |
| intercept | | 30.7091 | 0.9639 | 31.861 | <2×10^-16^ | 0.1449 | 3081 |
| age (at baseline) | | -0.0418 | 0.0176 | -2.383 | 0.0173 |  |  |
| t2d | | 13.6203 | 1.4981 | 9.092 | <2×10^-16^ |  |  |
| sex | | -1.3251 | 0.2611 | -5.074 | 4.12×10^-7^ |  |  |
| edu4* | | -1.0763 | 0.2319 | -4.641 | 3.62×10^-6^ |  |  |
| t2d:age | | -0.1925 | 0.0266 | -7.225 | 6.28×10^-13^ |  |  |
| t2d:sex | | 1.5213 | 0.4014 | 3.79 | 1.54×10^-4^ |  |  |
| Model 2: linear regression between standardized residual and global ancestry | | | | | | | |
| intercept | | -0.0638 | 0.0543 | -1.175 | 0.2402 | 0.0625 | 3084 |
| PNS | | 0.5923 | 0.0914 | 6.484 | 1.04×10^-10^ |  |  |
| EAS | | -0.6400 | 0.0726 | -8.817 | <2×10^-16^ |  |  |
| AFR | | 1.0777 | 0.6449 | 1.671 | 0.0948 |  |  |
|  | |  |  |  |  |  |  |
| Model 1A: linear mixed model between BMI and covariates, including nSES | | | | | | | |
| intercept | | 31.9467 | 1.0524 | 30.355 | <2×10^-16^ | 0.1574 | 2840 |
| age (at baseline) | | -0.0445 | 0.0185 | -2.399 | 0.0165 |  |  |
| t2d | | 12.9035 | 1.5541 | 8.303 | <2×10^-16^ |  |  |
| sex | | -1.4258 | 0.2766 | -5.155 | 2.72×10^-7^ |  |  |
| edu4* | | -0.6812 | 0.2483 | -2.743 | 0.0061 |  |  |
| nSES | (Q2 vs. Q1) | -0.5086 | 0.4023 | -1.264 | 0.2082 |  |  |
|  | (Q3 vs. Q1) | -1.4301 | 0.3862 | -3.703 | 2.95×10^-4^ |  |  |
|  | (Q4 vs. Q1) | -0.9863 | 0.3835 | -2.572 | 0.0112 |  |  |
|  | (Q5 vs. Q1) | -1.7015 | 0.3709 | -4.587 | 1.00×10^-5^ |  |  |
| t2d:age | | -0.1821 | 0.0276 | -6.606 | 4.69×10^-11^ |  |  |
| t2d:sex | | 1.4948 | 0.4171 | 3.584 | 3.45×10^-4^ |  |  |
| Model 2A: linear regression between standardized residual, including nSES, and global ancestry | | | | | | | |
| intercept | | -0.0331 | 0.0569 | -0.583 | 0.56 | 0.0516 | 2836 |
| NH | | 0.4974 | 0.0958 | 5.189 | 2.26×10^-7^ |  |  |
| EAS | | -0.6113 | 0.0761 | -8.034 | 1.37×10^-15^ |  |  |
| AFR | | 0.8537 | 0.6681 | 1.278 | 0.201 |  |  |

Model 1 models the non-genetic covariates according to the heuristic described in the **Methods**. The residual from model 1 is then inverse normalized and tested in model 2. Models 1A and 2A repeats the procedure but included quintiles of nSES levels in a mixed effect model (**Methods**); in this case, the R^2^ in Model 1A reported include both the fixed and the random effect. * edu4 was a binary variable created from the original categorical variable of education status by grouping levels 1,2,3 and coded 0, while education status level 4 was coded as 1. This was done because there were no significant associations between education levels 1 through 3 and BMI. See Supplemental Table 21 for description of these education levels.
