# Supplementary material for: The impact of global and local Polynesian genetic ancestry on complex traits in Native Hawaiians
Source: PLoS Genet. 2021 Feb 11;17(2):e1009273. doi: 10.1371/journal.pgen.1009273 (PMC7877570; doi:10.1371/journal.pgen.1009273)
Supplement: S2 Table — Model 1 models the non-genetic covariates according to the heuristic described in the Methods. The residual from model 1 is then inverse normalized and tested in model 2. The top panels were conducted in males only; the bottom in females only. See S21 Table for description of these education and cigarette smoking levels. (DOCX) [file pgen.1009273.s012.docx]

**S2 Table: Details of the association statistics of the covariates and global ancestries of WHR**.

| Model 1: linear regression between WHR and covariates in males | | | | | | | |
| --- | --- | --- | --- | --- | --- | --- | --- |
| variables | | estimate | std. error | t | p | R^2^ | df |
| intercept | | 0.8655 | 0.0207 | 41.88 | <2×10^-16^ | 0.1126 | 1150 |
| age | | -0.0002 | 0.0002 | -0.859 | 0.3905 |  |  |
| bmi | | 0.0037 | 0.0004 | 10.386 | <2×10^-16^ |  |  |
| edu | (2 vs. 1) | -0.0063 | 0.0080 | -0.784 | 0.4334 |  |  |
|  | (3 vs. 1) | -0.0097 | 0.0080 | -1.203 | 0.2291 |  |  |
|  | (4 vs. 1) | -0.0192 | 0.0083 | -2.321 | 0.0205 |  |  |
| cig | (1&2&3 vs. 4) | 0.0130 | 0.0042 | 3.071 | 0.0022 |  |  |
|  | (1&2&3 vs. 5) | 0.0150 | 0.0051 | 2.929 | 0.0035 |  |  |
|  | (1&2&3 vs. 6) | 0.0128 | 0.0071 | 1.796 | 0.0728 |  |  |
| Model 2: linear regression between standardized residual and global ancestry in males | | | | | | | |
| intercept | | 0.1613 | 0.0923 | 1.748 | 0.0808 | 0.0089 | 1155 |
| PNS | | -0.3592 | 0.1515 | -2.372 | 0.0179 |  |  |
| EAS | | -0.1358 | 0.1221 | -1.112 | 0.2664 |  |  |
| AFR | | 1.9487 | 1.2585 | 1.548 | 0.1218 |  |  |
|  | |  |  |  |  |  |  |
| Model 1: linear regression between WHR and covariates in females | | | | | | | |
| variables | | estimate | std. error | t | p | R^2^ | df |
| intercept | | 0.7832 | 0.0204 | 38.347 | <2×10^-16^ | 0.0638 | 1494 |
| age | | 0.0005 | 0.0003 | 1.964 | 0.0497 |  |  |
| bmi | | 0.0028 | 0.0003 | 8.597 | <2×10^-16^ |  |  |
| edu | (2 vs. 1) | -0.0272 | 0.0089 | -3.041 | 0.0024 |  |  |
|  | (3 vs. 1) | -0.0231 | 0.0090 | -2.556 | 0.0107 |  |  |
|  | (4 vs. 1) | -0.0296 | 0.0094 | -3.153 | 0.0017 |  |  |
| cig | (1&2&3 vs. 4) | 0.0083 | 0.0052 | 1.595 | 0.1108 |  |  |
|  | (1&2&3 vs. 5) | 0.0192 | 0.0076 | 2.517 | 0.0120 |  |  |
|  | (1&2&3 vs. 6) | 0.0048 | 0.0122 | 0.394 | 0.6934 |  |  |
| Model 2: linear regression between standardized residual and global ancestry in males | | | | | | | |
| intercept | | -0.1606 | 0.0780 | -2.06 | 0.0395 | 0.0050 | 1499 |
| PNS | | 0.2272 | 0.1374 | 1.653 | 0.0985 |  |  |
| EAS | | 0.2587 | 0.1051 | 2.462 | 0.0139 |  |  |
| AFR | | -0.1722 | 0.9257 | -0.186 | 0.8524 |  |  |

Model 1 models the non-genetic covariates according to the heuristic described in the **Methods**. The residual from model 1 is then inverse normalized and tested in model 2. The top panels were conducted in males only; the bottom in females only. See Supplemental Table 21 for description of these education and cigarette smoking levels.
