# Supplementary material for: The impact of global and local Polynesian genetic ancestry on complex traits in Native Hawaiians
Source: PLoS Genet. 2021 Feb 11;17(2):e1009273. doi: 10.1371/journal.pgen.1009273 (PMC7877570; doi:10.1371/journal.pgen.1009273)
Supplement: S3 Table — Model 1 models the non-genetic covariates according to the heuristic described in the Methods. The residual from model 1 is then inverse normalized and tested in model 2. (DOCX) [file pgen.1009273.s013.docx]

**S3 Table: Details of the association statistics of the covariates and global ancestries of fasting glucose.**

| Model 1: linear regression between glucose and covariates | | | | | | |
| --- | --- | --- | --- | --- | --- | --- |
| variables | estimate | std. error | t | p | R^2^ | df |
| intercept | 4.0461 | 0.1902 | 21.27 | <2x10^-16^ | 0.0216 | 1258 |
| age (at blood draw) | 0.0039 | 0.0024 | 1.608 | 0.1081 |  |  |
| bmi | 0.0170 | 0.0038 | 4.529 | 6.49x10^-6^ |  |  |
| sex | -0.0657 | 0.0381 | -1.723 | 0.0851 |  |  |
| Model 2: linear regression between standardized residual and global ancestry | | | | | | |
| Intercept | -0.0611 | 0.0810 | -0.754 | 0.451 | 0.0024 | 1258 |
| PNS | 0.0044 | 0.1436 | 0.031 | 0.976 |  |  |
| EAS | 0.1585 | 0.1125 | 1.409 | 0.159 |  |  |
| AFR | 1.2135 | 1.0259 | 1.183 | 0.237 |  |  |

Model 1 models the non-genetic covariates according to the heuristic described in the **Methods**. The residual from model 1 is then inverse normalized and tested in model 2.
