# Supplementary material for: The impact of global and local Polynesian genetic ancestry on complex traits in Native Hawaiians
Source: PLoS Genet. 2021 Feb 11;17(2):e1009273. doi: 10.1371/journal.pgen.1009273 (PMC7877570; doi:10.1371/journal.pgen.1009273)
Supplement: S4 Table — Model 1 models the non-genetic covariates according to the heuristic described in the Methods. The residual from model 1 is then inverse normalized and tested in model 2. (DOCX) [file pgen.1009273.s014.docx]

S4 Table: Details of the association statistics of the covariates and global ancestries of fasting insulin.

| Model 1: linear regression between ln(insulin) and covariates | | | | | | |
| --- | --- | --- | --- | --- | --- | --- |
| variables | estimate | std. error | T | p | R^2^ | df |
| intercept | 0.4118 | 0.1721 | 2.393 | 0.0169 | 0.1585 | 1265 |
| age (at blood draw) | -0.0012 | 0.0022 | -0.565 | 0.5719 |  |  |
| bmi | 0.0523 | 0.0034 | 15.4 | <2x10^-16^ |  |  |
| sex | 0.0751 | 0.0344 | 2.18 | 0.0294 |  |  |
| Model 2: linear regression between standardized residual and global ancestry | | | | | | |
| Intercept | -0.1160 | 0.0807 | -1.437 | 0.1508 | 0.0033 | 1265 |
| PNS | 0.2716 | 0.1433 | 1.896 | 0.0582 |  |  |
| EAS | 0.0138 | 0.1124 | 0.123 | 0.9021 |  |  |
| AFR | 0.7477 | 1.0244 | 0.73 | 0.4656 |  |  |

Model 1 models the non-genetic covariates according to the heuristic described in the **Methods**. The residual from model 1 is then inverse normalized and tested in model 2.
