# Supplementary material for: The impact of global and local Polynesian genetic ancestry on complex traits in Native Hawaiians
Source: PLoS Genet. 2021 Feb 11;17(2):e1009273. doi: 10.1371/journal.pgen.1009273 (PMC7877570; doi:10.1371/journal.pgen.1009273)
Supplement: S5 Table — Model 1 models the non-genetic covariates according to the heuristic described in the Methods. The residual from model 1 is then inverse normalized and tested in model 2. Models 1A and 2A repeats the procedure but included quintiles of nSES levels in a mixed effect model (Methods); in this case, the R2 in Model 1A reported include both the fixed and the random effect. (DOCX) [file pgen.1009273.s015.docx]

S5 Table: Details of the association statistics of the covariates and global ancestries of HDL.

| Model 1: linear regression between HDL and covariates | | | | | | | |
| --- | --- | --- | --- | --- | --- | --- | --- |
| variables | | estimate | std. error | t | p | R^2^ | df |
| intercept | | 29.3271 | 2.9464 | 9.954 | <2×10^-16^ | 0.0641 | 1700 |
| age (at blood draw) | | 0.1310 | 0.0452 | 2.898 | 0.0038 |  |  |
| sex | | 7.6327 | 0.7329 | 10.415 | <2x10^-16^ |  |  |
| Model 2: linear regression between standardized residual and global ancestry | | | | | | | |
| intercept | | 0.1489 | 0.0712 | 2.093 | 0.0365 | 0.0169 | 1699 |
| PNS | | -0.4715 | 0.1235 | -3.817 | 1.40x10^-4^ |  |  |
| EAS | | 0.1753 | 0.0967 | 1.813 | 0.0700 |  |  |
| AFR | | -1.4498 | 0.8777 | -1.652 | 0.0988 |  |  |
|  | |  |  |  |  |  |  |
| Model 1A: linear mixed model between HDL and covariates, including nSES | | | | | | | |
| intercept | | 25.6 | 3.332 | 7.682 | 3.29×10^-14^ | 0.0902 | 1555 |
| age (at blood draw) | | 0.1308 | 0.048 | 2.717 | 0.0067 |  |  |
| sex | | 7.665 | 0.770 | 9.96 | <2×10^-16^ |  |  |
| nSES | (Q2 vs. Q1) | 5.518 | 1.535 | 3.595 | 4.66×10^-4^ |  |  |
|  | (Q3 vs. Q1) | 2.89 | 1.474 | 1.96 | 0.0520 |  |  |
|  | (Q4 vs. Q1) | 3.208 | 1.448 | 2.215 | 0.0287 |  |  |
|  | (Q5 vs. Q1) | 5.922 | 1.394 | 4.248 | 4.37×10^-5^ |  |  |
| Model 2A: linear regression between standardized residual, including nSES, and global ancestry | | | | | | | |
| intercept | | 0.0787 | 0.0749 | 1.051 | 0.2935 | 0.0091 | 1551 |
| PNS | | -0.3027 | 0.1301 | -2.327 | 0.0201 |  |  |
| EAS | | 0.1725 | 0.1014 | 1.701 | 0.0891 |  |  |
| AFR | | -0.9393 | 0.9099 | -1.032 | 0.3021 |  |  |

Model 1 models the non-genetic covariates according to the heuristic described in the **Methods**. The residual from model 1 is then inverse normalized and tested in model 2. Models 1A and 2A repeats the procedure but included quintiles of nSES levels in a mixed effect model (**Methods**); in this case, the R^2^ in Model 1A reported include both the fixed and the random effect.
