# Supplementary material for: The impact of global and local Polynesian genetic ancestry on complex traits in Native Hawaiians
Source: PLoS Genet. 2021 Feb 11;17(2):e1009273. doi: 10.1371/journal.pgen.1009273 (PMC7877570; doi:10.1371/journal.pgen.1009273)
Supplement: S6 Table — Model 1 models the non-genetic covariates according to the heuristic described in the Methods. The residual from model 1 is then inverse normalized and tested in model 2. (DOCX) [file pgen.1009273.s016.docx]

S6 Table: Details of the association statistics of the covariates and global ancestries of LDL.

| Model 1: linear regression between LDL and covariates | | | | | | |
| --- | --- | --- | --- | --- | --- | --- |
| variables | estimate | std. error | t | p | R^2^ | df |
| intercept | 148.2936 | 7.1638 | 20.7 | <2×10^-16^ | 0.0045 | 1678 |
| age (at blood draw) | -0.0951 | 0.1098 | -0.866 | 0.3866 |  |  |
| sex | 4.6162 | 1.7807 | 2.592 | 0.0096 |  |  |
| Model 2: linear regression between rank-based inversed residual and global ancestry | | | | | | |
| intercept | -0.0434 | 0.0721 | -0.602 | 0.547 | 7.96×10^-4^ | 1677 |
| PNS | 0.0736 | 0.1252 | 0.588 | 0.557 |  |  |
| EAS | 0.0720 | 0.0981 | 0.734 | 0.463 |  |  |
| AFR | -0.5192 | 0.8890 | -0.584 | 0.559 |  |  |

Model 1 models the non-genetic covariates according to the heuristic described in the **Methods**. The residual from model 1 is then inverse normalized and tested in model 2.
