# Supplementary material for: The impact of global and local Polynesian genetic ancestry on complex traits in Native Hawaiians
Source: PLoS Genet. 2021 Feb 11;17(2):e1009273. doi: 10.1371/journal.pgen.1009273 (PMC7877570; doi:10.1371/journal.pgen.1009273)
Supplement: S7 Table — Model 1 models the non-genetic covariates according to the heuristic described in the Methods. The residual from model 1 is then inverse normalized and tested in model 2. * edu4 was a binary variable created from the original categorical variable of education status by grouping levels 1,2,3 and coded 0, while education status level 4 was coded as 1. This was done because there were no significant associations between education levels 1 through 3 and BMI. (DOCX) [file pgen.1009273.s017.docx]

S7 Table: Details of the association statistics of the covariates and global ancestries of TG.

| Model 1: linear regression between ln(TG) and covariates | | | | | | |
| --- | --- | --- | --- | --- | --- | --- |
| variables | estimate | std. error | t | p | R^2^ | df |
| intercept | 4.7329 | 0.1044 | 45.32 | <2×10^-16^ | 0.0026 | 1691 |
| age (at blood draw) | 0.0002 | 0.0016 | 0.116 | 0.908 |  |  |
| sex | -0.0187 | 0.0256 | -0.732 | 0.4641 |  |  |
| edu4* | -0.0577 | 0.0293 | -1.969 | 0.0492 |  |  |
| Model 2: linear regression between rank-based inversed residual and global ancestry | | | | | | |
| intercept | -0.0972 | 0.0377 | -2.578 | 0.0100 | 0.0055 | 1691 |
| PNS | 0.1387 | 0.0654 | 2.119 | 0.0342 |  |  |
| EAS | 0.1426 | 0.0511 | 2.791 | 0.0053 |  |  |
| AFR | 0.1639 | 0.4635 | 0.354 | 0.7237 |  |  |

Model 1 models the non-genetic covariates according to the heuristic described in the **Methods**. The residual from model 1 is then inverse normalized and tested in model 2. * edu4 was a binary variable created from the original categorical variable of education status by grouping levels 1,2,3 and coded 0, while education status level 4 was coded as 1. This was done because there were no significant associations between education levels 1 through 3 and BMI.
