# Supplementary material for: The impact of global and local Polynesian genetic ancestry on complex traits in Native Hawaiians
Source: PLoS Genet. 2021 Feb 11;17(2):e1009273. doi: 10.1371/journal.pgen.1009273 (PMC7877570; doi:10.1371/journal.pgen.1009273)
Supplement: S8 Table — Model 1 models the non-genetic covariates according to the heuristic described in the Methods. The residual from model 1 is then inverse normalized and tested in model 2. (DOCX) [file pgen.1009273.s018.docx]

S8 Table: Details of the association statistics of the covariates and global ancestries of total cholesterol.

| Model 1: linear regression between TC and covariates | | | | | | |
| --- | --- | --- | --- | --- | --- | --- |
| variables | estimate | std. error | t | p | R^2^ | df |
| intercept | 205.7 | 7.671 | 26.816 | <2×10^-16^ | 0.0190 | 1702 |
| age (at blood draw) | 0.0057 | 0.1176 | 0.048 | 0.961 |  |  |
| sex | 10.94 | 1.907 | 5.733 | 1.16×10^-8^ |  |  |
| Model 2: linear regression between rank-based inversed residual and global ancestry | | | | | | |
| intercept | -0.0337 | 0.0716 | -0.471 | 0.6375 | 0.0046 | 1701 |
| PNS | -0.0441 | 0.1243 | -0.355 | 0.7228 |  |  |
| EAS | 0.2072 | 0.0972 | 2.132 | 0.0331 |  |  |
| AFR | -0.8084 | 0.8832 | -0.915 | 0.3602 |  |  |

Model 1 models the non-genetic covariates according to the heuristic described in the **Methods**. The residual from model 1 is then inverse normalized and tested in model 2.
