# Supplementary material for: The impact of global and local Polynesian genetic ancestry on complex traits in Native Hawaiians
Source: PLoS Genet. 2021 Feb 11;17(2):e1009273. doi: 10.1371/journal.pgen.1009273 (PMC7877570; doi:10.1371/journal.pgen.1009273)
Supplement: S9 Table — Model 1 models the non-genetic covariates according to the heuristic described in the Methods. Model 2 then includes global ancestries in addition to the significant covariates. * edu4 was a binary variable created from the original categorical variable of education status by grouping levels 1,2,3 and coded 0, while education status level 4 was coded as 1. This was done because there were no significant associations between education levels 1 through 3 and obesity. Model 3 included quintiles of nSES levels in a mixed effect model. (DOCX) [file pgen.1009273.s019.docx]

S9 Table: Details of the association statistics of the covariates and global ancestries of obesity.

| Model 1: logistic regression based on covariates | | | | | | |
| --- | --- | --- | --- | --- | --- | --- |
| variables | | estimate | std. error | z | p | df |
| intercept | | -0.9591 | 0.4966 | -1.932 | 0.0534 | 3083 |
| age (at baseline) | | -0.0139 | 0.0093 | -1.496 | 0.1346 |  |
| t2d | | 4.1860 | 0.6733 | 6.217 | 5.06×10^-10^ |  |
| edu4* | | -0.2234 | 0.1079 | -2.07 | 0.0385 |  |
| t2d:age | | -0.0526 | 0.0124 | -4.233 | 2.31×10^-5^ |  |
| Model 2: logistic regression between obesity and covariates | | | | | | |
| intercept | | -1.0136 | 0.5225 | -1.94 | 0.0524 | 3080 |
| PNS | | 1.2164 | 0.2349 | 5.178 | 2.24×10^-7^ |  |
| EAS | | -1.3596 | 0.2073 | -6.559 | 5.40×10^-11^ |  |
| AFR | | 1.3181 | 1.5591 | 0.845 | 0.3979 |  |
| age (at baseline) | | -0.0170 | 0.0095 | -1.791 | 0.0734 |  |
| t2d | | 4.4103 | 0.6901 | 6.391 | 1.65×10^-10^ |  |
| edu4* | | -0.0789 | 0.1113 | -0.709 | 0.4782 |  |
| t2d:age | | -0.0562 | 0.0127 | -4.415 | 1.01×10^-5^ |  |
| Model 3: logistic mixed model including nSES | | | | | | |
| Intercept | | -1.6383 | 0.2000 | -8.193 | 2.55×10^-16^ | 2827 |
| PNS | | 1.0774 | 0.2470 | 4.361 | 1.29×10^-5^ |  |
| EAS | | -1.3105 | 0.2146 | -6.105 | 1.03×10^-9^ |  |
| AFR | | 1.2973 | 1.5884 | 0.817 | 0.4141 |  |
| age (baseline) | | -0.0196 | 0.0099 | -1.972 | 0.0487 |  |
| t2d | | 1.2979 | 0.0978 | 13.275 | <2×10^-16^ |  |
| edu4* | | -0.0317 | 0.1174 | -0.27 | 0.7872 |  |
| nSES | (Q2 vs. Q1) | -0.1483 | 0.1664 | -0.891 | 0.3730 |  |
|  | (Q3 vs. Q1) | -0.3517 | 0.1648 | -2.134 | 0.0328 |  |
|  | (Q4 vs. Q1) | -0.1163 | 0.1602 | -0.726 | 0.4680 |  |
|  | (Q5 vs. Q1) | -0.4175 | 0.1598 | -2.613 | 0.0090 |  |
| t2d:age | | -0.0523 | 0.0132 | -3.956 | 7.63×10^-5^ |  |

Model 1 models the non-genetic covariates according to the heuristic described in the **Methods**. Model 2 then includes global ancestries in addition to the significant covariates. * edu4 was a binary variable created from the original categorical variable of education status by grouping levels 1,2,3 and coded 0, while education status level 4 was coded as 1. This was done because there were no significant associations between education levels 1 through 3 and obesity. Model 3 included quintiles of nSES levels in a mixed effect model.
