# Supplementary material for: The impact of global and local Polynesian genetic ancestry on complex traits in Native Hawaiians
Source: PLoS Genet. 2021 Feb 11;17(2):e1009273. doi: 10.1371/journal.pgen.1009273 (PMC7877570; doi:10.1371/journal.pgen.1009273)
Supplement: S10 Table — Model 1 models the non-genetic covariates according to the heuristic described in the Methods. Model 2 then includes global ancestries in addition to the significant covariates. Model 3 included quintiles of nSES levels in a mixed effect model. * edu3 was a ternary variable created from the original categorical variable of education status by grouping levels 1 and 2. This was done because there were no significant associations between education levels 1 and 2 with T2D. (DOCX) [file pgen.1009273.s020.docx]

S10 Table: Details of the association statistics of the covariates and global ancestries of Type-2 Diabetes.

| Model 1: logistic regression based on covariates | | | | | | | |
| --- | --- | --- | --- | --- | --- | --- | --- |
| variables | | | estimate | std. error | z | p | Df |
| intercept | | | -7.4892 | 0.4224 | -17.729 | <2×10^-16^ | 3047 |
| age (at baseline) | | | 0.0644 | 0.0054 | 11.814 | <2×10^-16^ |  |
| bmi | | | 0.1317 | 0.0079 | 16.754 | <2×10^-16^ |  |
| edu3* | 3 vs (1 & 2) | | -0.3022 | 0.0919 | -3.289 | 0.0010 |  |
|  | 4 vs (1 & 2) | | -0.3631 | 0.1043 | -3.482 | 4.97×10^-4^ |  |
| Model 2: logistics regression between type 2 diabetes and covariates | | | | | | | |
| intercept | | | -8.4590 | 0.4556 | -18.566 | <2×10^-16^ | 3045 |
| PNS | | | 0.8209 | 0.2179 | 3.767 | 1.65×10^-4^ |  |
| EAS | | | 1.1765 | 0.1738 | 6.769 | 1.30×10^-11^ |  |
| AFR | | | 0.3603 | 1.5188 | 0.237 | 0.8125 |  |
| age (at baseline) | | | 0.0660 | 0.0055 | 11.972 | <2×10^-16^ |  |
| bmi | | | 0.1384 | 0.0082 | 16.946 | <2×10^-16^ |  |
| edu3* | 3 vs (1 & 2) | | -0.3059 | 0.0930 | -3.289 | 0.0010 |  |
|  | 4 vs (1 & 2) | | -0.3666 | 0.1066 | -3.44 | 0.0006 |  |
| Model 3: logistic mixed model including nSES | | | | | | | |
| Intercept | | | -0.5990 | 0.1826 | -3.281 | 0.0010 | 2827 |
| PNS | | | 0.7575 | 0.2271 | 3.336 | 8.51×10^-4^ |  |
| EAS | | | 1.1569 | 0.1774 | 6.522 | 6.92×10^-11^ |  |
| AFR | | | 0.2535 | 1.5109 | 0.168 | 0.8668 |  |
| age (at baseline) | | | 0.0625 | 0.0057 | 10.932 | <2×10^-16^ |  |
| bmi | | | 0.1326 | 0.0084 | 15.738 | <2×10^-16^ |  |
| edu3* | | 3 vs (1 & 2) | -0.2943 | 0.0958 | -3.073 | 0.0021 |  |
|  |  | 4 vs (1 & 2) | -0.3317 | 0.1110 | -2.987 | 0.0028 |  |
| nSES | | (Q2 vs. Q1) | -0.2271 | 0.1485 | -1.529 | 0.1262 |  |
|  |  | (Q3 vs. Q1) | -0.1418 | 0.1445 | -0.981 | 0.3265 |  |
|  |  | (Q4 vs. Q1) | -0.2876 | 0.1432 | -2.008 | 0.0447 |  |
|  |  | (Q5 vs. Q1) | -0.2808 | 0.1389 | -2.021 | 0.0433 |  |

Model 1 models the non-genetic covariates according to the heuristic described in the **Methods**. Model 2 then includes global ancestries in addition to the significant covariates. Model 3 included quintiles of nSES levels in a mixed effect model. * edu3 was a ternary variable created from the original categorical variable of education status by grouping levels 1 and 2. This was done because there were no significant associations between education levels 1 and 2 with T2D.
