# Supplementary material for: The impact of global and local Polynesian genetic ancestry on complex traits in Native Hawaiians
Source: PLoS Genet. 2021 Feb 11;17(2):e1009273. doi: 10.1371/journal.pgen.1009273 (PMC7877570; doi:10.1371/journal.pgen.1009273)
Supplement: S11 Table — Model 1 models the non-genetic covariates according to the heuristic described in the Methods. Model 2 then includes global ancestries in addition to the significant covariates. Model 3 included quintiles of nSES levels in a mixed effect model. * edu3 was a ternary variable created from the original categorical variable of education status by grouping levels 1 and 2. This was done because there were no significant associations between education levels 1 and 2 with heart failure. (DOCX) [file pgen.1009273.s021.docx]

S11 Table: Details of the association statistics of the covariates and global ancestries of heart failure.

| Model 1: logistics regression based on covariates | | | | | | |
| --- | --- | --- | --- | --- | --- | --- |
| variables | | estimate | std. error | z | p | df |
| intercept | | -9.0826 | 0.5881 | -15.444 | <2×10^-16^ | 2218 |
| age (at baseline) | | 0.1012 | 0.0076 | 13.306 | <2×10^-16^ |  |
| bmi | | 0.0928 | 0.0093 | 9.957 | <2×10^-16^ |  |
| sex | | -0.4895 | 0.1063 | -4.607 | 4.09×10^-6^ |  |
| edu3* | 3 vs (1 & 2) | -0.2746 | 0.1210 | -2.269 | 0.0233 |  |
|  | 4 vs (1 & 2) | -0.3991 | 0.1426 | -2.798 | 0.0051 |  |
| Model 2: logistics regression between heart failure and covariates | | | | | | |
| intercept | | -9.5113 | 0.6263 | -15.186 | <2×10^-16^ | 2215 |
| PNS | | 1.0465 | 0.2831 | 3.697 | 2.18×10^-4^ |  |
| EAS | | 0.2528 | 0.2338 | 1.081 | 0.2797 |  |
| AFR | | 3.4653 | 1.8375 | 1.886 | 0.0593 |  |
| age (at baseline) | | 0.1009 | 0.0077 | 13.195 | <2×10^-16^ |  |
| bmi | | 0.0882 | 0.0096 | 9.189 | <2×10^-16^ |  |
| sex | | -0.4909 | 0.1069 | -4.592 | 4.39×10^-6^ |  |
| edu3* | 3 vs (1 & 2) | -0.2432 | 0.1217 | -1.998 | 0.0457 |  |
|  | 4 vs (1 & 2) | -0.3227 | 0.1444 | -2.234 | 0.0255 |  |
| Model 3: logistic mixed model including nSES | | | | | | |
| intercept | | -1.1414 | 0.2632 | -4.336 | 1.45×10^-5^ | 2055 |
| PNS | | 1.0201 | 0.3024 | 3.374 | 7.41×10^-4^ |  |
| EAS | | 0.2775 | 0.2458 | 1.129 | 0.2588 |  |
| AFR | | 3.4056 | 1.8889 | 1.803 | 0.0714 |  |
| age (at baseline) | | 0.1018 | 0.0083 | 12.325 | <2×10^-16^ |  |
| bmi | | 0.0899 | 0.0102 | 8.84 | <2×10^-16^ |  |
| sex | | -0.5090 | 0.1120 | -4.546 | 5.46×10^-6^ |  |
| edu3* | 3 vs (1 & 2) | -0.2291 | 0.1282 | -1.788 | 0.0738 |  |
|  | 4 vs (1 & 2) | -0.3298 | 0.1532 | -2.153 | 0.0314 |  |
| nSES | (Q2 vs. Q1) | -0.2241 | 0.2128 | -1.053 | 0.2923 |  |
|  | (Q3 vs. Q1) | -0.3938 | 0.2123 | -1.855 | 0.0636 |  |
|  | (Q4 vs. Q1) | -0.4033 | 0.2102 | -1.918 | 0.0551 |  |
|  | (Q5 vs. Q1) | -0.3256 | 0.2030 | -1.604 | 0.1087 |  |

Model 1 models the non-genetic covariates according to the heuristic described in the **Methods**. Model 2 then includes global ancestries in addition to the significant covariates. Model 3 included quintiles of nSES levels in a mixed effect model. * edu3 was a ternary variable created from the original categorical variable of education status by grouping levels 1 and 2. This was done because there were no significant associations between education levels 1 and 2 with heart failure.
