# Supplementary material for: The impact of global and local Polynesian genetic ancestry on complex traits in Native Hawaiians
Source: PLoS Genet. 2021 Feb 11;17(2):e1009273. doi: 10.1371/journal.pgen.1009273 (PMC7877570; doi:10.1371/journal.pgen.1009273)
Supplement: S12 Table — Model 1 models the non-genetic covariates according to the heuristic described in the Methods. Model 2 then includes global ancestries in addition to the significant covariates. * edu4 was a binary variable created from the original categorical variable of education status by grouping levels 1,2,3 and coded 0, while education status level 4 was coded as 1. This was done because there were no significant associations between education levels 1 through 3 and hyperlipidemia. (DOCX) [file pgen.1009273.s022.docx]

S12 Table: Details of the association statistics of the covariates and global ancestries of hyperlipidemia.

| Model 1: logistics regression based on covariates | | | | | |
| --- | --- | --- | --- | --- | --- |
| variables | estimate | std. error | z | p | df |
| intercept | -2.0533 | 0.3836 | -5.353 | 8.64×10^-8^ | 2222 |
| age (at baseline) | 0.0528 | 0.0070 | 7.526 | 5.23×10^-14^ |  |
| edu4* | 0.2674 | 0.1144 | 2.338 | 0.0194 |  |
| Model 2: logistics regression between hyperlipidemia and covariates | | | | | |
| intercept | -2.3231 | 0.4066 | -5.713 | 1.11×10^-8^ | 2219 |
| PNS | 0.0652 | 0.2473 | 0.264 | 0.7920 |  |
| EAS | 0.6973 | 0.2012 | 3.465 | 5.30×10^-4^ |  |
| AFR | 0.9841 | 1.7462 | 0.564 | 0.5731 |  |
| age (at baseline) | 0.0535 | 0.0070 | 7.599 | 2.98×10^-14^ |  |
| edu4* | 0.2529 | 0.1158 | 2.184 | 0.0290 |  |

Model 1 models the non-genetic covariates according to the heuristic described in the **Methods**. Model 2 then includes global ancestries in addition to the significant covariates. * edu4 was a binary variable created from the original categorical variable of education status by grouping levels 1,2,3 and coded 0, while education status level 4 was coded as 1. This was done because there were no significant associations between education levels 1 through 3 and hyperlipidemia.
