# Supplementary material for: The impact of global and local Polynesian genetic ancestry on complex traits in Native Hawaiians
Source: PLoS Genet. 2021 Feb 11;17(2):e1009273. doi: 10.1371/journal.pgen.1009273 (PMC7877570; doi:10.1371/journal.pgen.1009273)
Supplement: S13 Table — Model 1 models the non-genetic covariates according to the heuristic described in the Methods. Model 2 then includes global ancestries in addition to the significant covariates. (DOCX) [file pgen.1009273.s023.docx]

**S13 Table: Details of the association statistics of the covariates and global ancestries of hypertension.**

| Model 1: logistic regression based on covariates | | | | | |
| --- | --- | --- | --- | --- | --- |
| variables | estimate | std. error | z | p | df |
| intercept | -5.2619 | 0.5108 | -10.302 | <2×10^-16^ | 2235 |
| age (at baseline) | 0.0859 | 0.0076 | 11.269 | <2×10^-16^ |  |
| bmi | 0.0537 | 0.0092 | 5.812 | 6.17×10^-9^ |  |
| Model 2: logistics regression between hypertension and covariates | | | | | |
| intercept | -5.8251 | 0.5313 | -10.963 | <2×10^-16^ | 2232 |
| PNS | 0.3842 | 0.2570 | 1.495 | 0.135 |  |
| EAS | 0.8783 | 0.2053 | 4.277 | 1.89×10^-5^ |  |
| AFR | -0.9459 | 1.7337 | -0.546 | 0.585 |  |
| age (at baseline) | 0.0865 | 0.0077 | 11.296 | <2×10^-16^ |  |
| bmi | 0.0584 | 0.0095 | 6.129 | 8.85×10^-10^ |  |

Model 1 models the non-genetic covariates according to the heuristic described in the **Methods**. Model 2 then includes global ancestries in addition to the significant covariates.
