# Supplementary material for: The impact of global and local Polynesian genetic ancestry on complex traits in Native Hawaiians
Source: PLoS Genet. 2021 Feb 11;17(2):e1009273. doi: 10.1371/journal.pgen.1009273 (PMC7877570; doi:10.1371/journal.pgen.1009273)
Supplement: S14 Table — Model 1 models the non-genetic covariates according to the heuristic described in the Methods. Model 2 then includes global ancestries in addition to the significant covariates. * edu3 was a ternary variable created from the original categorical variable of education status by grouping levels 1 and 2. This was done because there were no significant associations between education levels 1 and 2 with ischemic heart disease. (DOCX) [file pgen.1009273.s024.docx]

S14 Table: Details of the association statistics of the covariates and global ancestries for ischemic heart disease.

| Model 1: logistics regression based on covariates | | | | | | |
| --- | --- | --- | --- | --- | --- | --- |
| variables | | estimate | std. error | z | p | df |
| intercept | | -6.5801 | 0.4983 | -13.204 | <2×10^-16^ | 2218 |
| age (at baseline) | | 0.0942 | 0.0068 | 13.862 | <2×10^-16^ |  |
| bmi | | 0.0476 | 0.0083 | 5.764 | 8.22×10^-9^ |  |
| sex | | -0.4739 | 0.0932 | -5.087 | 3.63×10^-7^ |  |
| edu3* | 3 vs (1 & 2) | -0.2965 | 0.1066 | -2.782 | 0.0054 |  |
|  | 4 vs (1 & 2) | -0.3636 | 0.1221 | -2.979 | 0.0029 |  |
| Model 2: logistics regression between ischemic heart diseases and covariates | | | | | | |
| intercept | | -6.7066 | 0.5205 | -12.886 | <2×10^-16^ | 2215 |
| PNS | | 0.2881 | 0.2481 | 1.161 | 0.2457 |  |
| EAS | | 0.1445 | 0.1970 | 0.733 | 0.4633 |  |
| AFR | | -0.7074 | 1.7560 | -0.403 | 0.6871 |  |
| age (at baseline) | | 0.0939 | 0.0068 | 13.795 | <2×10^-16^ |  |
| bmi | | 0.0471 | 0.0085 | 5.535 | 3.11×10^-8^ |  |
| sex | | -0.4685 | 0.0934 | -5.015 | 5.31×10^-7^ |  |
| edu3* | 3 vs (1 & 2) | -0.2894 | 0.1069 | -2.707 | 0.0068 |  |
|  | 4 vs (1 & 2) | -0.3473 | 0.1235 | -2.813 | 0.0049 |  |

Model 1 models the non-genetic covariates according to the heuristic described in the **Methods**. Model 2 then includes global ancestries in addition to the significant covariates. * edu3 was a ternary variable created from the original categorical variable of education status by grouping levels 1 and 2. This was done because there were no significant associations between education levels 1 and 2 with ischemic heart disease.
