# Supplementary material for: The impact of global and local Polynesian genetic ancestry on complex traits in Native Hawaiians
Source: PLoS Genet. 2021 Feb 11;17(2):e1009273. doi: 10.1371/journal.pgen.1009273 (PMC7877570; doi:10.1371/journal.pgen.1009273)
Supplement: S15 Table — Model 1 models the non-genetic covariates according to the heuristic described in the Methods. Model 2 then includes global ancestries in addition to the significant covariates. (DOCX) [file pgen.1009273.s025.docx]

**S15 Table: Details of the association statistics of the covariates and global ancestries for stroke and transient ischemic attacks.**

| Model 1: logistics regression based on covariates | | | | | |
| --- | --- | --- | --- | --- | --- |
| variables | estimate | std. error | z | p | df |
| intercept | -8.8502 | 0.6655 | -13.298 | <2×10^-16^ | 2235 |
| age (at baseline) | 0.1056 | 0.0087 | 12.084 | <2×10^-16^ |  |
| bmi | 0.0344 | 0.0111 | 3.087 | 0.0020 |  |
| Model 2: logistics regression between stroke and transient ischemic attacks and covariates | | | | | |
| intercept | -8.9589 | 0.7007 | -12.785 | <2×10^-16^ | 2232 |
| PNS | 0.3612 | 0.3370 | 1.072 | 0.2839 |  |
| EAS | 0.0719 | 0.2738 | 0.263 | 0.7928 |  |
| AFR | 2.1347 | 2.1583 | 0.989 | 0.3226 |  |
| age (at baseline) | 0.1053 | 0.0088 | 11.991 | <2×10^-16^ |  |
| bmi | 0.0320 | 0.0115 | 2.781 | 0.0054 |  |

Model 1 models the non-genetic covariates according to the heuristic described in the **Methods**. Model 2 then includes global ancestries in addition to the significant covariates.
