# Supplementary material for: The impact of global and local Polynesian genetic ancestry on complex traits in Native Hawaiians
Source: PLoS Genet. 2021 Feb 11;17(2):e1009273. doi: 10.1371/journal.pgen.1009273 (PMC7877570; doi:10.1371/journal.pgen.1009273)
Supplement: S16 Table — For traits we analyzed in Table 2 accounting for the impact due to neighborhood SES, we adopted the same model (S1, S5 and S9–S11 Tables) but dichotomized the nSES variable, grouping quintiles 1–3 into the “low” nSES group, and quintiles 4–5 into the “high” nSES group, and included the interaction terms between each of the ancestry component and the dichotomized nSES variable. Mixed effect modeling was then performed. The effect on BMI associated with each ancestry in the low and high nSES groups are provided, as well as the P-value for the interaction terms (Phet). We observed no significant interaction between ancestry and dichotomized nSES measure. (DOCX) [file pgen.1009273.s026.docx]

**S16 Table: Stratified analysis and interaction between ancestry components and neighborhood SES measures.**

| Trait | PNS | | | EAS | | | AFR | | |
| --- | --- | --- | --- | --- | --- | --- | --- | --- | --- |
|  | β (low) | β (high) | P_het_ | β (low) | β (high) | P_het_ | β (low) | β (high) | P_het_ |
| BMI | 0.554 | 0.542 | 0.951 | -0.632 | -0.623 | 0.956 | 0.518 | 1.680 | 0.397 |
| HDL | -0.331 | -0.392 | 0.816 | 0.0701 | 0.224 | 0.45 | -1.567 | -0.857 | 0.698 |
| Obesity | 1.191 | 1.030 | 0.743 | -1.246 | -1.375 | 0.763 | 0.0095 | 3.891 | 0.238 |
| T2D | 0.485 | 1.112 | 0.163 | 0.919 | 1.398 | 0.172 | 0.567 | -0.523 | 0.725 |
| HF | 0.635 | 1.554 | 0.128 | 0.0459 | 0.538 | 0.315 | 3.808 | 2.216 | 0.702 |

For traits we analyzed in **Table 2** accounting for the impact due to neighborhood SES, we adopted the same model (**S1, S5, S9-S11 Tables**) but dichotomized the nSES variable, grouping quintiles 1-3 into the “low” nSES group, and quintiles 4-5 into the “high” nSES group, and included the interaction terms between each of the ancestry component and the dichotomized nSES variable. Mixed effect modeling was then performed. The effect on BMI associated with each ancestry in the low and high nSES groups are provided, as well as the P-value for the interaction terms (P_het_). We observed no significant interaction between ancestry and dichotomized nSES measure.
