# Supplementary material for: The impact of global and local Polynesian genetic ancestry on complex traits in Native Hawaiians
Source: PLoS Genet. 2021 Feb 11;17(2):e1009273. doi: 10.1371/journal.pgen.1009273 (PMC7877570; doi:10.1371/journal.pgen.1009273)
Supplement: S17 Table — Model 1 models the non-genetic covariates according to the heuristic described in the Methods, except for sex. The residual from model 1 was then inverse normalized and tested in model 2, which includes global ancestries, sex, and interactions between global ancestries and sex. * edu4 was a binary variable created from the original categorical variable of education status by grouping levels 1,2,3 and coded 0, while education status level 4 was coded as 1. This was done because there were no significant associations between education levels 1 through 3 and BMI. (DOCX) [file pgen.1009273.s027.docx]

S17 Table: Model of association between global ancestry and BMI, including interaction with sex.

| Model 1: linear regression between BMI and covariates | | | | | | |
| --- | --- | --- | --- | --- | --- | --- |
| variables | estimate | std. error | t | p | R^2^ | df |
| intercept | 29.8203 | 0.9514 | 31.345 | <2×10^-16^ | 0.1377 | 3083 |
| age (at baseline) | -0.0398 | 0.0176 | -2.258 | 0.0240 |  |  |
| t2d | 14.6333 | 1.4769 | 9.908 | <2×10^-16^ |  |  |
| age:t2d | -0.1950 | 0.0267 | -7.298 | 3.69×10^-13^ |  |  |
| edu4^*^ | -1.0224 | 0.2324 | -4.4 | 1.15×10^-5^ |  |  |
| Model 2: linear regression between rank-based inversed residual and global ancestry | | | | | | |
| intercept | 0.0714 | 0.0834 | 0.856 | 0.392 | 0.0741 | 3080 |
| PNS | 0.4685 | 0.1351 | 3.469 | 5.29×10^-4^ |  |  |
| EAS | -0.5489 | 0.1110 | -4.946 | 7.97×10^-7^ |  |  |
| AFR | 1.7034 | 1.1484 | 1.483 | 0.138 |  |  |
| sex | -0.2559 | 0.1097 | -2.332 | 0.0198 |  |  |
| PNS:sex | 0.2513 | 0.1827 | 1.375 | 0.169 |  |  |
| EAS:sex | -0.1637 | 0.1464 | -1.119 | 0.263 |  |  |
| AFR:sex | -0.8465 | 1.3846 | -0.611 | 0.541 |  |  |

Model 1 models the non-genetic covariates according to the heuristic described in the **Methods**, except for sex. The residual from model 1 was then inverse normalized and tested in model 2, which includes global ancestries, sex, and interactions between global ancestries and sex. * edu4 was a binary variable created from the original categorical variable of education status by grouping levels 1,2,3 and coded 0, while education status level 4 was coded as 1. This was done because there were no significant associations between education levels 1 through 3 and BMI.
