# Supplementary material for: The impact of global and local Polynesian genetic ancestry on complex traits in Native Hawaiians
Source: PLoS Genet. 2021 Feb 11;17(2):e1009273. doi: 10.1371/journal.pgen.1009273 (PMC7877570; doi:10.1371/journal.pgen.1009273)
Supplement: S18 Table — Model testing was performed in the same manner as the global analysis with BMI (S1 Table), except for stratifying based on T2D disease status. Model column provided the final model with association coefficients. * edu4 was a binary variable created from the original categorical variable of education status by grouping levels 1,2,3 and coded 0, while education status level 4 was coded as 1. This was done because there were no significant associations between education levels 1 through 3 and BMI. (DOCX) [file pgen.1009273.s028.docx]

**S18 Table: Stratified analysis of association between global genetic ancestry and BMI among T2D cases and controls.**

| Strata: Type 2 diabetes | | | | | | |
| --- | --- | --- | --- | --- | --- | --- |
| Model 1: linear regression between BMI and covariates | | | | | | |
| variables | estimate | std. error | t | p | R^2^ | df |
| intercept | 44.3084 | 1.2509 | 35.421 | <2×10^-16^ | 0.0868 | 1290 |
| age (at baseline) | -0.2341 | 0.0216 | -10.859 | <2×10^-16^ |  |  |
| sex | 0.1986 | 0.3272 | 0.607 | 0.544 |  |  |
| edu4* | -1.0289 | 0.4105 | -2.506 | 0.0123 |  |  |
| Model 2: linear regression between rank-based inversed residual and global ancestry | | | | | | |
| Intercept | 0.1326 | 0.0946 | 1.402 | 0.161 | 0.0590 | 1290 |
| PNS | 0.2371 | 0.1492 | 1.588 | 0.112 |  |  |
| EAS | -0.8096 | 0.1196 | -6.769 | 1.96×10^-11^ |  |  |
| AFR | 0.6460 | 1.0207 | 0.633 | 0.527 |  |  |
|  | | | | | | |
| Strata: Non-type 2 diabetes | | | | | | |
| Model 1: linear regression between BMI and covariates | | | | | | |
| variables | estimate | std. error | t | p | R^2^ | df |
| intercept | 30.7277 | 0.9192 | 33.43 | <2×10^-16^ | 0.0251 | 1790 |
| age (at baseline) | -0.0420 | 0.0167 | -2.523 | 0.0117 |  |  |
| sex | -1.3266 | 0.2473 | -5.364 | 9.19×10^-8^ |  |  |
| edu4* | -1.1036 | 0.2756 | -4.005 | 6.47×10^-5^ |  |  |
| Model 2: linear regression between rank-based inversed residual and global ancestry | | | | | | |
| intercept | -0.1963 | 0.0664 | -2.959 | 0.0031 | 0.0730 | 1790 |
| PNS | 0.8745 | 0.1173 | 7.453 | 1.41×10^-13^ |  |  |
| EAS | -0.5347 | 0.0917 | -5.832 | 6.48×10^-9^ |  |  |
| AFR | 1.3586 | 0.8293 | 1.638 | 0.1016 |  |  |

Model testing was performed in the same manner as the global analysis with BMI (**S1 Table**), except for stratifying based on T2D disease status. Model column provided the final model with association coefficients. * edu4 was a binary variable created from the original categorical variable of education status by grouping levels 1,2,3 and coded 0, while education status level 4 was coded as 1. This was done because there were no significant associations between education levels 1 through 3 and BMI.
