# Supplementary material for: The impact of global and local Polynesian genetic ancestry on complex traits in Native Hawaiians
Source: PLoS Genet. 2021 Feb 11;17(2):e1009273. doi: 10.1371/journal.pgen.1009273 (PMC7877570; doi:10.1371/journal.pgen.1009273)
Supplement: S19 Table — Model 1 models the non-genetic covariates according to the heuristic described in the Methods, except for type-2 diabetes status. The residual from model 1 was then inverse normalized and tested in model 2, which includes global ancestries, type-2 diabetes status, and interactions between global ancestries and type-2 diabetes status. * edu4 was a binary variable created from the original categorical variable of education status by grouping levels 1,2,3 and coded 0, while education status level 4 was coded as 1. This was done because there were no significant associations between education levels 1 through 3 and BMI. (DOCX) [file pgen.1009273.s029.docx]

S19 Table: Model of association between global ancestry and BMI, including interaction with type-2 diabetes.

| Model 1: linear regression between BMI and covariates | | | | | | |
| --- | --- | --- | --- | --- | --- | --- |
| variables | estimate | std. error | t | p | R^2^ | df |
| intercept | 34.1237 | 0.7376 | 46.264 | <2×10^-16^ | 0.0212 | 3401 |
| age (at baseline) | -0.0832 | 0.0131 | -6.378 | 2.10×10^-10^ |  |  |
| Sex | -0.7239 | 0.2006 | -3.608 | 3.13×10^-4^ |  |  |
| edu4^*^ | -1.2995 | 0.2347 | -5.538 | 3.29×10^-8^ |  |  |
| Model 2: linear regression between rank-based inversed residual and global ancestry | | | | | | |
| intercept | -0.4586 | 0.0628 | -7.307 | 3.46×10^-13^ | 0.1738 | 3080 |
| PNS | 0.8330 | 0.1110 | 7.506 | 7.95×10^-14^ |  |  |
| EAS | -0.5164 | 0.0867 | -5.954 | 2.91×10^-9^ |  |  |
| AFR | 1.1126 | 0.7844 | 1.418 | 0.1562 |  |  |
| t2d | 0.9937 | 0.1087 | 9.139 | <2×10^-16^ |  |  |
| PNS:t2d | -0.6338 | 0.1787 | -3.546 | 0.0004 |  |  |
| EAS:t2d | -0.1846 | 0.1419 | -1.301 | 0.1933 |  |  |
| AFR:t2d | -0.5000 | 1.2382 | -0.404 | 0.6864 |  |  |

Model 1 models the non-genetic covariates according to the heuristic described in the **Methods**, except for type-2 diabetes status. The residual from model 1 was then inverse normalized and tested in model 2, which includes global ancestries, type-2 diabetes status, and interactions between global ancestries and type-2 diabetes status. * edu4 was a binary variable created from the original categorical variable of education status by grouping levels 1,2,3 and coded 0, while education status level 4 was coded as 1. This was done because there were no significant associations between education levels 1 through 3 and BMI.
