# Supplementary material for: The impact of global and local Polynesian genetic ancestry on complex traits in Native Hawaiians
Source: PLoS Genet. 2021 Feb 11;17(2):e1009273. doi: 10.1371/journal.pgen.1009273 (PMC7877570; doi:10.1371/journal.pgen.1009273)
Supplement: S20 Table — Reported P-value, associated trait, and mapped genes were provided by the GWAS catalog. Allele frequencies were either calculated from the imputed data of the 178 reference MEC Native Hawaiian individuals with estimated PNS ancestry > 90%, or obtained from 1000 Genomes Project. Frequencies were reported with respect to the minor allele in the Native Hawaiians, given in parenthesis next to the Native Hawaiian frequency estimates. (DOCX) [file pgen.1009273.s030.docx]

**S20 Table: Variants within the admixture signal region that were reported to be associated with the tested or related traits in GWAS catalog.**

| SNP ID | Chr | Pos (hg19) | Reported P-value | Associated Trait | Mapped Gene | Allele Frequencies | | | |
| --- | --- | --- | --- | --- | --- | --- | --- | --- | --- |
|  |  |  |  |  |  | MEC-NH | EUR | EAS | AFR |
| rs79976124 | 6 | 66618657 | 2x10^-6^ | type 2 diabetes | NUFIP1P, ADH5P4 | 0.198 (A) | 0.288 | 0.0784 | 0.0024 |
| rs10498828 | 6 | 65533066 | 9x10^-6^ | type 2 diabetes | EYS | 0.164 (T) | 0.218 | 0.109 | 0.128 |

Reported P-value, associated trait, and mapped genes were provided by the GWAS catalog [4]. Allele frequencies were either calculated from the imputed data of the 178 reference MEC Native Hawaiian individuals with estimated PNS ancestry > 90%, or obtained from 1000 Genomes Project. Frequencies were reported with respect to the minor allele in the Native Hawaiians, given in parenthesis next to the Native Hawaiian frequency estimates.
