# Supplementary material for: The impact of global and local Polynesian genetic ancestry on complex traits in Native Hawaiians
Source: PLoS Genet. 2021 Feb 11;17(2):e1009273. doi: 10.1371/journal.pgen.1009273 (PMC7877570; doi:10.1371/journal.pgen.1009273)
Supplement: S21 Table — Allele frequencies were either calculated from the imputed data of the 178 reference MEC Native Hawaiian individuals with estimated PNS ancestry > 90%, or obtained from 1000 Genomes Project (EUR, EAS, and AFR; https://www.internationalgenome.org/1000-genomes-browsers/) or the Genome Asia data (Oceania and Southeast Asia; https://browser.genomeasia100k.org/). Frequencies were reported with respect to the derived allele, given in parenthesis next to the Native Hawaiian frequency estimates. (DOCX) [file pgen.1009273.s031.docx]

**S21 Table: Allele frequencies across populations for the most strongly associated variant in chr6 for T2D in single variant association test.**

| SNP ID | Chr | Pos (hg19) | Derived Allele Frequencies | | | | | |
| --- | --- | --- | --- | --- | --- | --- | --- | --- |
|  |  |  | MEC-NH | EUR | EAS | AFR | Oceania | Southeast Asia |
| rs370140172 | 6 | 66205761 | 0.243 (C) | 0 | 0.009 | 0 | 0.020 | 0.0116 |

Allele frequencies were either calculated from the imputed data of the 178 reference MEC Native Hawaiian individuals with estimated PNS ancestry > 90%, or obtained from 1000 Genomes Project (EUR, EAS, and AFR; <https://www.internationalgenome.org/1000-genomes-browsers/>) or the Genome Asia data (Oceania and Southeast Asia; <https://browser.genomeasia100k.org/>). Frequencies were reported with respect to the derived allele, given in parenthesis next to the Native Hawaiian frequency estimates.
