# Supplementary material for: The impact of global and local Polynesian genetic ancestry on complex traits in Native Hawaiians
Source: PLoS Genet. 2021 Feb 11;17(2):e1009273. doi: 10.1371/journal.pgen.1009273 (PMC7877570; doi:10.1371/journal.pgen.1009273)
Supplement: S22 Table — We attempted to replicate the association of rs370140172 and nine other proxies showing the strongest single-variant associations with a cross-sectional population based study of Samoans recruited from Independent Samoa (Methods). EAF, effect allele frequency in Samoans. BETA and SE refers to the effect size and standard errors, respectively, from the logistic mixed model association tests in the Samoan cohort. P-val (Samoa) and P-val (MEC-NH) provide the p-value from the logistic mixed model association tests in the Samoan cohort and MEC Native Hawaiian cohort, respectively. (DOCX) [file pgen.1009273.s032.docx]

**S22 Table: Association results to T2D in 2,852 Samoan Replication Cohort.**

| rsID | Pos (hg19, chr6) | Effect Allele | Other Allele | EAF | BETA | SE | P-val (Samoa) | P-val  (MEC-NH) |
| --- | --- | --- | --- | --- | --- | --- | --- | --- |
| rs13213141 | 64295122 | A | G | 0.216 | -0.002 | 0.091 | 0.986 | 1.770x10^-4^ |
| rs62415478 | 65472417 | C | G | 0.239 | 0.015 | 0.086 | 0.858 | 1.034x10^-4^ |
| rs60268597 | 65473673 | A | G | 0.237 | 0.020 | 0.086 | 0.813 | 2.012x10^-4^ |
| rs62415480 | 65475772 | T | C | 0.237 | 0.019 | 0.086 | 0.830 | 2.012x10^-4^ |
| rs72648371 | 65507941 | T | C | 0.197 | 0.030 | 0.093 | 0.743 | 1.927x10^-4^ |
| rs374288303 | 66156770 | C | T | 0.087 | 0.021 | 0.132 | 0.873 | 1.698x10^-5^ |
| rs369186009 | 66197658 | C | G | NA | NA | NA | NA | 4.209x10^-5^ |
| rs370140172 | 66205761 | C | T | 0.087 | 0.025 | 0.132 | 0.852 | 1.248x10^-5^ |
| rs79261478 | 66348532 | T | C | 0.087 | 0.011 | 0.133 | 0.934 | 2.612x10^-5^ |
| rs75566215 | 66358487 | C | T | 0.087 | 0.011 | 0.133 | 0.934 | 2.258x10^-5^ |

We attempted to replicate the association of rs370140172 and nine other proxies showing the strongest single-variant associations with a cross-sectional population based study of Samoans recruited from Independent Samoa (**Methods**). EAF, effect allele frequency in Samoans. BETA and SE refers to the effect size and standard errors, respectively, from the logistic mixed model association tests in the Samoan cohort. P-val (Samoa) and P-val (MEC-NH) provide the p-value from the logistic mixed model association tests in the Samoan cohort and MEC Native Hawaiian cohort, respectively.
