# Supplementary material for: The impact of global and local Polynesian genetic ancestry on complex traits in Native Hawaiians
Source: PLoS Genet. 2021 Feb 11;17(2):e1009273. doi: 10.1371/journal.pgen.1009273 (PMC7877570; doi:10.1371/journal.pgen.1009273)
Supplement: S23 Table — To evaluate the impact of recombination map on local ancestry inference, we used the 1000 Genomes AMR population. Following the same procedure used for Native Hawaiians, we identified through unsupervised ADMIXTURE analysis 49 Peruvian (PEL) and 3 Mexican (MEX) individuals from 1000 Genomes as having > 80% Native American ancestry. We then inferred local ancestry using RFMix in 71 HapMap3 MEX individuals using the constructed reference panel of 99 CEU, 108 YRI, and 52 NA individuals from 1000 Genomes. We used three recombination map in the local ancestry inference: a HapMap2 pooled recombination map, a mis-specified African-American map, and a constant map that assumes a constant rate of 1cM/Mb across the genome. We compared in pairwise fashion the concordance of inferred ancestry across common variants between runs, and calculated concordance rate as the sum of the diagonal of the contingency table. Across all comparisons, even when using a constant rate map, the concordance rate is extremely high (0.987, 0.981, and 0.981 for the comparisons of default vs. AA map, default to constant rate map, and constant rate to AA map, respectively), suggesting that the choice of recombination map does not strongly impact the local ancestry inference using RFMix. (DOCX) [file pgen.1009273.s033.docx]

**S23 Table: Local ancestry inference using RFMix is robust to the choice of recombination map.**

| Genetic map 1  Genetic map 2 | | AA Map | | |
| --- | --- | --- | --- | --- |
|  |  | CEU | NA | YRI |
| Default Map | CEU | 0.4629 | 0.0048 | 0.0013 |
|  | NA | 0.0052 | 0.4803 | 0.0004 |
|  | YRI | 0.0010 | 0.0005 | 0.0436 |

| Genetic map 1  Genetic map 2 | | Constant Map | | |
| --- | --- | --- | --- | --- |
|  |  | CEU | NA | YRI |
| Default Map | CEU | 0.4603 | 0.0078 | 0.0009 |
|  | NA | 0.0069 | 0.4787 | 0.0004 |
|  | YRI | 0.0022 | 0.0008 | 0.0420 |

| Genetic map 1  Genetic map 2 | | AA Map | | |
| --- | --- | --- | --- | --- |
|  |  | CEU | NA | YRI |
| Constant Map | CEU | 0.4605 | 0.0065 | 0.0023 |
|  | NA | 0.0079 | 0.4785 | 0.0008 |
|  | YRI | 0.0008 | 0.0005 | 0.0421 |

To evaluate the impact of recombination map on local ancestry inference, we used the 1000 Genomes AMR population. Following the same procedure used for Native Hawaiians, we identified through unsupervised ADMIXTURE analysis 49 Peruvian (PEL) and 3 Mexican (MEX) individuals from 1000 Genomes as having > 80% Native American ancestry. We then inferred local ancestry using RFMix in 71 HapMap3 MEX individuals using the constructed reference panel of 99 CEU, 108 YRI, and 52 NA individuals from 1000 Genomes. We used three recombination map in the local ancestry inference: a HapMap2 pooled recombination map, a mis-specified African-American map [5], and a constant map that assumes a constant rate of 1cM / Mb across the genome. We compared in pairwise fashion the concordance of inferred ancestry across common variants between runs, and calculated concordance rate as the sum of the diagonal of the contingency table. Across all comparisons, even when using a constant rate map, the concordance rate is extremely high (0.987, 0.981, and 0.981 for the comparisons of default vs. AA map, default to constant rate map, and constant rate to AA map, respectively), suggesting that the choice of recombination map does not strongly impact the local ancestry inference using RFMix.
