# Supplementary material for: The impact of global and local Polynesian genetic ancestry on complex traits in Native Hawaiians
Source: PLoS Genet. 2021 Feb 11;17(2):e1009273. doi: 10.1371/journal.pgen.1009273 (PMC7877570; doi:10.1371/journal.pgen.1009273)
Supplement: S24 Table — These traits were studied in PAGE consortium and we thus follow the same criteria and transformation. (DOCX) [file pgen.1009273.s034.docx]

S24 Table: phenotype inclusion and transformation for metabolic and quantitative cardiovascular traits.

| Trait | Inclusion criteria and transformation |
| --- | --- |
| Glucose | Remove individuals with non-fasting glucose, with measurement greater than 7 mmol/L, who are pregnant, or have type-2 diabetes. |
| Insulin | Remove individuals with non-fasting insulin, with measurement of glucose greater than 7 mmol/L, who are pregnant, or have type 2. Insulin is then log-transformed. |
| HDL | Medication adjustments were made per table below, based on the approach reported in PAGE consortium. If multiple medications were reported, only the correction factor with largest effect was applied. Exclude individuals who are pregnant or who had not fasted for at least 8 hrs before blood draw. |
| LDL | Medication adjustments were made per table below, based on the approach reported in PAGE consortium. If multiple medications were reported, only the correction factor with largest effect was applied. Exclude individuals who are pregnant or who had not fasted for at least 8 hrs before blood draw, or individuals with measured LDL greater than 400 mg/dL. |
| TG | Medication adjustments were made per table below, based on the approach reported in PAGE consortium. If multiple medications were reported, only the correction factor with largest effect was applied. Adjusted TG was then log-transformed. Exclude individuals who are pregnant or who had not fasted for at least 8 hrs before blood draw, or individuals with measured TG greater than 3000 mg/dL. |
| TC | Medication adjustments were made per table below, based on the approach reported in PAGE consortium. If multiple medications were reported, only the correction factor with largest effect was applied. Exclude individuals who are pregnant or who had not fasted for at least 8 hrs before blood draw. |
| Type-2 diabetes | Remove among diabetes cases those individuals who are pregnant or who have been diagnosed as type 1 diabetes. Remove cases with age under 20 and controls with glucose over 7 mmol/L. |

| Medication  Trait | Fibrates | Statins | Bile acid sequestrants | Niacin | Cholesterol absorption inhibitors |
| --- | --- | --- | --- | --- | --- |
| HDL | -5.9 | -2.3 | -1.9 | -9.9 | 0 |
| LDL | +40.1 | +49.9 | +40.5 | +24.7 | +40.5 |
| TG | +57.1 | +18.4 | 0 | +89.4 | 0 |
| TC | +46.1 | +52.1 | 0 | +34.6 | +40.5 |

These traits were studied in PAGE consortium and we thus follow the same criteria and transformation.
