# Supplementary material for: The impact of global and local Polynesian genetic ancestry on complex traits in Native Hawaiians
Source: PLoS Genet. 2021 Feb 11;17(2):e1009273. doi: 10.1371/journal.pgen.1009273 (PMC7877570; doi:10.1371/journal.pgen.1009273)
Supplement: S25 Table — Summary statistics reported after exclusion and transformation as described in S20 Table. For biomarkers (glucose, insulin, HDL, LDL, TG, and TC), a subset of participants were invited after cohort entry. Thus there is an age at baseline and an age at blood draw. (DOCX) [file pgen.1009273.s035.docx]

S25 Table: Descriptive summary statistics of the traits and covariates analyzed.

| Quantitative traits | | | | |
| --- | --- | --- | --- | --- |
| Traits | | Sample size | mean | s.d. |
| BMI/m^2^·kg^-1^ | | 3427 | 28.90 | 5.86 |
| Waist-to-hip ratio (WHR) | | 2699 | 0.91 | 0.0072 |
| Glucose/mmol·L^-1^ | | 1693 | 4.98 | 1.28 |
| Insulin/pmol·L^-1^ | | 1700 | 8.31 | 7.48 |
| HDL/mg·dL^-1^ | Before adjustment | 1715 | 42.35 | 15.55 |
|  | After adjustment | 1711 | 41.61 | 15.60 |
| LDL/mg·dL^-1^ | Before adjustment | 1705 | 127.81 | 36.92 |
|  | After adjustment | 1701 | 144.33 | 36.88 |
| TG/mg·dL^-1^ | Before adjustment | 1717 | 122.421 | 80.27 |
|  | After adjustment | 1713 | 129.88 | 81.63 |
| TC/mg·dL^-1^ | Before adjustment | 1717 | 194.28 | 39.48 |
|  | After adjustment | 1713 | 211.59 | 39.71 |
|  | |  |  |  |
| Categorical traits | | | | |
| Traits | | Total size | Level | |
|  |  |  | 0 = undiagnosed | 1 = diagnosed |
| T2D | | 3109 | 1799 | 1310 |
| Obesity | | 3427 | 2612 | 815 |
| HF | | 2239 | 1683 | 556 |
| HYPERL | | 2239 | 657 | 1582 |
| HYPERT | | 2239 | 639 | 1600 |
| IHD | | 2239 | 1351 | 888 |
| STROKE_TIA | | 2239 | 1933 | 306 |
|  | |  |  |  |
| Quantitative covariates | | | | |
| Covariate | | Sample size | Mean | s.d. |
| Age at baseline | | 3428 | 54.26 | 7.67 |
| Age at blood draw | | 1717 | 64.08 | 8.09 |
|  | |  |  |  |
| Categorical covariates | | | | |
| Covariate | | Total size | Level | Number |
| Sex | | 3428 | 1 = Male | 1512 |
|  |  |  | 2 = Female | 1916 |
| Education (edu) | | 3406 | 1: ≤8^th^ grade | 222 |
|  |  |  | 2: high school | 1190 |
|  |  |  | 3: some college or vocational school | 1177 |
|  |  |  | 4: college graduates | 817 |
| Cigs_per_day (cig) | | 3381 | 1: 0 cigarettes smoked per day | 1483 |
|  |  |  | 2: ≤5 cigarettes smoked per day | 219 |
|  |  |  | 3: 6-10 cigarettes smoked per day | 429 |
|  |  |  | 4: 11-20 cigarettes smoked per day | 731 |
|  |  |  | 5: 21-30 cigarettes smoked per day | 353 |
|  |  |  | 6: > 31 cigarettes smoked per day | 166 |
|  |  |  |  |  |

Summary statistics reported after exclusion and transformation as described in S20 Table. For biomarkers (glucose, insulin, HDL, LDL, TG, and TC), a subset of the participants was invited after cohort entry. Thus there is an age at baseline and an age at blood draw.
